# Chromatin remodelers HELLS and UHRF1 mediate the epigenetic deregulation of genes that drive retinoblastoma tumor progression

**DOI:** 10.18632/oncotarget.2468

**Published:** 2014-10-13

**Authors:** Claudia A. Benavente, David Finkelstein, Dianna A. Johnson, Jean-Christophe Marine, Ruth Ashery-Padan, Michael A. Dyer

**Affiliations:** ^1^ Department of Developmental Neurobiology, St. Jude Children’s Research Hospital, Memphis, TN, USA; ^2^ Department of Computational Biology, St. Jude Children’s Research Hospital, Memphis, TN, USA; ^3^ Department of Ophtalmology, The University of Tennessee Health Science Center, Memphis, TN, USA; ^4^ Department of Anatomy and Neurobiology, The University of Tennessee Health Science Center, Memphis, TN, USA; ^5^ Laboratory for Molecular Cancer Biology, Center for the Biology of Disease, VIB, Leuven, Belgium; ^6^ Department of Human Molecular Genetics and Biochemistry, Tel-Aviv University, Tel Aviv, Israel; ^7^ Howard Hughes Medical Institute, Chevy Chase, MD, USA

**Keywords:** Rb, E2F, HELLS, UHRF1, retinoblastoma, cancer

## Abstract

The retinoblastoma (Rb) family of proteins are key regulators of cell cycle exit during development and their deregulation is associated with cancer. Rb is critical for normal retinal development and germline mutations lead to retinoblastoma making retinae an attractive system to study Rb family signaling. Rb coordinates proliferation and differentiation through the E2f family of transcription factors, a critical interaction for the role of Rb in retinal development and tumorigenesis. However, whether the roles of the different E2fs are interchangeable in controlling development and tumorigenesis in the retina or if they have selective functions remains unknown. In this study, we found that E2f family members play distinct roles in the development and tumorigenesis. In *Rb*;*p107*-deficient retinae, E2f1 and E2f3 inactivation rescued tumor formation but only E2f1 rescued the retinal development phenotype. This allowed the identification of key target genes for Rb/E2f family signaling contributing to tumorigenesis and those contributing to developmental defects. We found that *Sox4* and *Sox11* genes contribute to the developmental phenotype and *Hells* and *Uhrf1* contribute to tumorigenesis. Using orthotopic human xenografts, we validated that upregulation of HELLS and UHRF1 is essential for the tumor phenotype. Also, these epigenetic regulators are important for the regulation of SYK.

## INTRODUCTION

The Rb protein and its two related family members (p107 and p130) regulate gene expression through interactions with E2F/DP heterodimeric transcription factors [1]. There are 6 E2F family members that bind to the Rb family (activator E2Fs: E2F1, E2F2 and E2F3a and repressor E2Fs: E2F3b, E2F4, and E2F5) and 3 DP family members (TFDP1, TFDP2, and TFDP3) that form heterodimers with these E2Fs [1] and show preferential binding to Rb, p107, or p130. While Rb, p107, and p130 show overlapping but distinct preferences for particular E2F family members to regulate transcription, it has been proposed that they may shift depending on the cellular context. For example, some cells express different constellations of Rb or E2F family members and this can lead to distinct regulation [2]. Or, when a gene is inactivated or overexpressed, the Rb/E2Fs may shift their binding preferences [3]. Beyond these differences, there are also distinct mechanisms of action. Rb proteins can directly regulate genes that control cell cycle exit, but they can also regulate the expression of genes that control cell fate specification and differentiation through mechanisms including chromatin remodeling and epigenetic control [4]. A major question in the field is how Rb/E2F interactions control proliferation and tumor suppression in coordination with cellular differentiation events.

The retina is a good system to study this question because of the role of Rb in tumorigenesis and development. One of the clearest connections between *RB1* gene inactivation and tumorigenesis is the retina. Almost all retinoblastomas have *RB1* gene inactivation and *RB1* gene inactivation is sufficient to promote retinoblastoma [5]. *RB1* also plays a role in retinal development. Mouse models with all combinations of Rb family knocked-out have been analyzed at several stages of retinal development to test for proliferation, cell death and differentiation [6, 7]. Rb loss is characterized by defects of rod photoreceptor terminal differentiation followed by cell death and disruption of horizontal cell synaptogenesis [6]. Rb, p107, and p130 are not interchangeable in the developing retina. For example, even though p107 is overexpressed in the absence of Rb, p107 cannot prevent the rod photoreceptor defect associated with Rb loss [7, 8]. Unlike humans, loss of Rb alone in the developing mouse retina is insufficient to initiate tumorigenesis due to compensation by p107, for which genetic mouse models of retinoblastoma utilize the loss of Rb with another Rb family member [9, 10].

In previous studies, Bremner’s group showed that inactivating *E2f1*, but not *E2f2* or *E2f3*, rescues most of the retinal differentiation defects seen in Rb deficient retinae, including rod differentiation and function [11]. Furthermore, only loss of E2f1 significantly reduced tumor development in *Rb*;*p107* deficient mice [12]. Together, these data suggest that inadequate regulation of Rb/E2F signaling substantially contributes to the phenotypic consequences of Rb deficiency. However, the unique or overlapping roles of Rb/E2F signaling in retinal development and retinoblastoma remain unknown.

In this study, we generated a series of genetically engineered mouse models to study the unique and overlapping roles of the E2fs in retinal development and retinoblastoma. We found that retinal differentiation defects and tumorigenesis following loss of the Rb family occurs as two independent events that rely on distinct E2f family members. The data generated from these models was used to identify downstream targets that contribute to tumorigenesis and development. We found that members of the SoxC transcription factor family, in particular Sox4 and Sox11, may be important for maintaining in proper retinal development. We also found that Uhrf1 (ubiquitin-like, containing PHD and RING finger domains 1) and Hells (helicase, lymphoid specific) proteins are overexpressed in retinoblastoma and may be responsible for the epigenetic changes seen in retinoblastoma and required for tumor survival. Furthermore, we showed that upregulation of HELLS is linked to the epigenetic activation of the *SYK* gene (spleen tyrosine kinase), previously described to be key for human retinoblastoma survival [5].

## RESULTS

### Rb Regulates Tumorigenesis Through Activator E2f1 and E2f3

**Figure 1:**
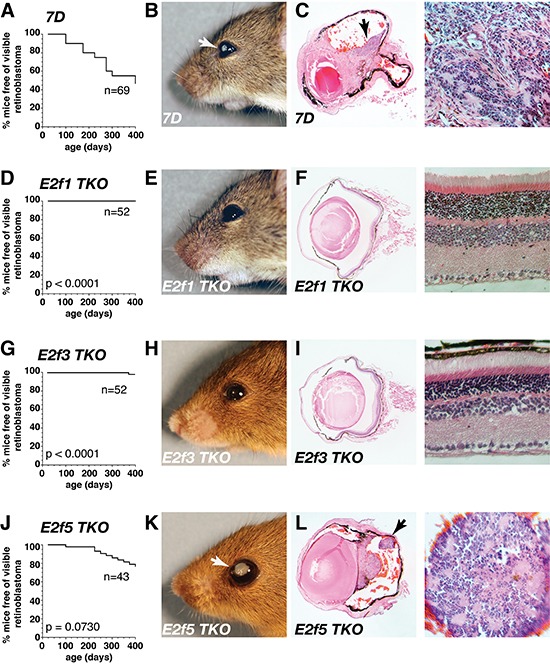
E2f1 and E2f3 inhibit tumorigenesis on a retinoblastoma-sensitized background **(A, D, G, J)** Kaplan–Meier curve showing the time to first observation of externally visible retinoblastoma. These times were significantly increased in **(D)** *Chx10-Cre;Rb^lox/lox^;p107^−/−^;E2f1^−/−^* (*E2f1 TKO*) and **(G)** *Chx10-Cre;Rb^lox/lox^;p107^−/−^;E2f3^lox/lox^* (*E2f3* TKO), but not **(J)** *Chx10-Cre;Rb^lox/lox^;p107^−/−^; E2f5^lox/lox^* (*E2f5 TKO*) relative to **(A)** *Chx10-Cre;Rb^lox/lox^;p107^−/−^ (7D)* littermates. **(B, E, H, K)** Representative **(B)** *7D* and **(K)** *E2f5 TKO* mice with aggressive retinoblastoma and 12-month-old **(E)** *E2f1 TKO* and **(H)** *E2f3 TKO* tumor-free mice. **(C, F, I, L)** Hematoxylin and eosin stain in whole eyes. **(C)** *7D* and **(L)** *E2f5 TKO* where tumor is visible (purple) with characteristic rosettes (higher magnification), and **(F)** *E2f1 TKO* and **(I)** *E2f3 TKO* at P370 showing no evidence of tumor.

Previous studies have shown that loss of *E2f1* rescues retinal differentiation and retinoblastoma formation, in Rb-deficient mice [12]. To determine if loss of other E2f family members expressed in the retina can prevent tumorigenesis or rescue the developmental defect in the *Rb*;*p107* deficient mice, we developed *Chx10-Cre;Rb^Lox/Lox^;p107^−/−^;E2f1^−/−^* (E2f1 TKO), *Chx10-Cre;Rb^Lox/Lox^;p107^−/−^;E2f3^Lox/Lox^* (E2f3 TKO), *Chx10-Cre;Rb^Lox/Lox^;p107^−/−^;E2f4^−/−^* (E2f4 TKO), and *Chx10-Cre;Rb^Lox/Lox^;p107^−/−^;E2f5^Lox/Lox^* (E2f5 TKO) triple-knockout mice. Previous studies have shown that approximately 50% of *Chx10-Cre;Rb^Lox/Lox^;p107^−/−^* (7D) mice develop retinoblastoma by 12 months of age [13]. To characterize the tumor incidence in our E2f TKO mouse models, we monitored mice weekly for up to 400 days of age or until advanced tumor burden required euthanasia. 46% (32/69) of the 7D mice developed tumors by 400 days old (Fig. 1A-C) but none of the 52 E2f1 TKO mice developed tumors (Fig. 1D-F). Similarly, only 1 of the 52 (2%) E2f3 TKOP mice developed tumors in the same timeframe (Fig. 1G-I). In contrast, 11 of the 43 (26%) E2f5 TKO mice developed retinoblastoma in 400 days (Fig. 1J-L) and this was not significantly different from the 7D strain (p=0.07). E2f4 TKO mice failed to thrive past the first few days of life.

To compare the cellular features of the 7D and E2f5 TKO tumors, we performed TEM analysis on 3 tumors from each strain. Previous analyses of the morphological and neuroanatomical features of mouse and human retinoblastomas have shown tightly packed clusters of cells and rosettes, dense core vesicles, apical tight junctions, mitochondrial-filled inner segment regions with cilia, and basally located synaptic ribbons and vesicles [13, 14]. E2f5 TKO tumors had all of the differentiation features of 7D and human retinoblastomas with processes and junctions, tightly packed tumor cells with areas of plexus, and dense core vesicles (Fig. S1). These data are consistent with previous characterization of cellular features of mouse retinoblastoma showing that the cellular features are indistinguishable across 6 different genotypes with different penetrance and tumor progression [13].

### Rb Regulates Rod Cell Fate Specification Through E2f1

**Figure 2:**
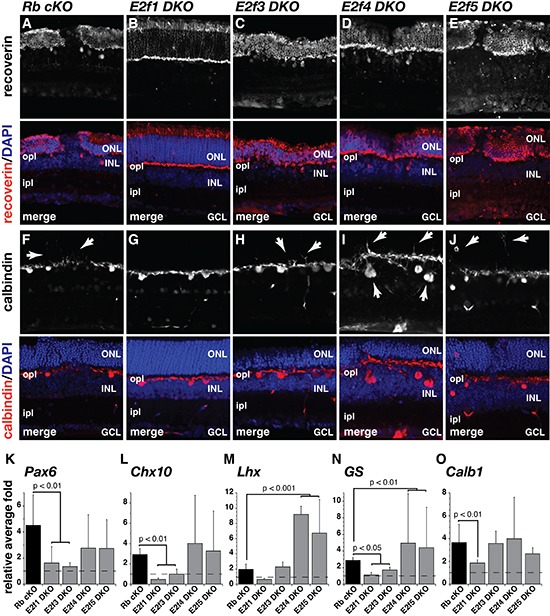
E2f1 restores proper retinogenesis in an Rb-null background **(A-E)** Recoverin immunostaining (red) labeled photoreceptors in the ONL and DAPI nuclear counterstain (blue) showed that the ONL, containing photoreceptors, is thinner in patches of **(A)** *Chx10-Cre;Rb^lox/lox^* (*Rb cKO*), **(C)** *Chx10-Cre;Rb^lox/lox^;E2f3^lox/lox^* (*E2f3 DKO*), **(D)** *Chx10-Cre;Rb^lox/lox^;E2f4^−/−^* (*E2f4 DKO*), and **(E)** *Chx10-Cre;Rb^lox/lox^;E2f5^lox/lox^* (*E2f5 DKO*) retinae at P21 but not **(B)** *Chx10-Cre;Rb^lox/lox^;E2f1^−/−^* (*E2f1 DKO*). **(F-J)** Calbindin immunostaining (red) labeled a subset of amacrine and horizontal cell bodies in the INL, as well as their respective synaptic processes in the IPL and OPL, respectively. In **(F)** *Rb cKO*, (H) *E2f3 DKO*, **(I)** *E2f4 DKO*, and **(J)** *E2f5 DKO* retina, calbindin immunopositive horizontal cell processes extended into the ONL (arrows) but not in **(G)** *E2f1 DKO*. Nuclei were counterstained with DAPI (blue). **(K-O)** Real-time RT-PCR analysis showed several genes overexpressed in *Rb cKO* retinae are restored back to wild type levels in *E2f1 DKO* or *E2f3 DKO* retinae but not *E2f4 DKO or E2f5 DKO*. All data are normalized to their wild type or control littermates (n=5). ONL, outer nuclear layer; INL, inner nuclear layer; OPL, outer plexiform layer; GCL, ganglion cell layer; ipl, inner plexiform layer; opl, outer plexiform layer.

In addition to its role in tumorigenesis, Rb1 is required for rod photoreceptor differentiation in the murine retina [6, 9, 15, 16]. A recent report has shown that inactivation of *E2f1* but not *E2f3* can restore rod differentiation in Rb-deficient retinae [12]. To extend those previous analyses, we generated *Chx10-Cre;Rb^Lox/Lox^; E2f1^−/−^* (E2f1 DKO), *Chx10-Cre; Rb^Lox/Lox^;E2f3^Lox/Lox^* (E2f3 DKO), *Chx10-Cre;Rb^Lox/Lox^;E2f4^−/−^* (E2f4 DKO), and *Chx10-Cre;Rb^Lox/Lox^;E2f5^Lox/Lox^* (E2f5 DKO) mice and harvested retinae at 3 different stages of retinal development (P14, P21 and P365). We performed immunostaining using 12 different antibodies for cell type specific markers, qPCR using 20 different genes and TEM analysis (see Supplemental Information). The *Rb1*-dependent rod differentiation defect was only rescued by inactivation of *E2f1* in our analysis. This is evident by the uniform layer and thickness of the outer nuclear layer (ONL) in the E2f1 DKO retinae, compared to the mosaic disruption and thinning of the ONL observed in Rb cKO, E2F3 DKO, E2f4 DKO and E2f5 DKO retinae (Fig. 2A-E). Previous studies have shown that the defect in rod differentiation in the *Rb1*-deficient retinae can lead to perturbations in outer plexiform layer (OPL) synaptogenesis and ectopic horizontal cell neurites extending into the ONL [6]. This phenotype was also rescued by inactivation of *E2f1* suggesting that the timing of photoreceptor differentiation and synaptogenesis was also rescued in the E2f1 DKO mouse retinae (Fig. 2F-J). The qPCR data provided independent validation of the histological analysis, showing a reduction in the expression levels of progenitor and interneuron cell markers (*Pax6*, *Chx10*, and *G.S.*) in E2f1 DKO and E2f3 DKO retinae but not E2f4 DKO and E2f5 DKO when compared to Rb cKO mRNA expression levels (Fig. 2K-O). Interestingly, we found that glutathione synthetase (*GS*) and Nestin (*Nes1*), genes expressed in Müller glia, are significantly upregulated in E2f4 DKO (5.0 ± 5.9 and 11.3 ± 3.5, respectively. p < 0.01) and E2f5 DKO retinae (4.4 ± 4.8 and 7.2 ± 5.6, respectively. p < 0.01), which is significantly higher than the upregulation seen in Rb loss alone (2.9 ± 0.3 for *GS* and 1.6 ± 0.4 for *Nes1*, Fig. 2N and S2). In addition, E2f4 DKO and E2f5 DKO retinae also show a significant increase in the expression of the horizontal cell gene marker *Lhx* (9.19 ± 11.04 and 5.8 ± 4.7, respectively. p < 0.001, Fig. 2M), while markers of other internuclear layer neurons are not significantly changed from the levels expressed by Rb loss alone (Fig. S2). These observations are interesting in the context of the genesis of retinoblastoma, retinae with persistent expression of progenitor and interneuron cell markers appear to be are more permissive to tumorigenesis. On the other hand, failure to terminally differentiate photoreceptors does not appear to contribute to the tumorigenic phenotype as E2f3 DKO mice have defects in the ONL, but E2f3 TKO mice do not form tumors.

To confirm our observations, we performed TEM analysis of retinae at P14, P21 and adult stages. TEM of E2f1 DKO mice show that there are no substantial differences between these and E2F1-deficient retinae. On the other hand, E2f3 DKO, E2f4 DKO and E2f5 DKO retinae show extensive dysmorphia including, reduced thickness of the outer nuclear layer (ONL) and collapse of the outer plexiform layer (OPL) (Fig. S3 and data not shown). In addition, there were rare cells in the ONL of the Rb cKO, E2f3 DKO, E2f4 DKO and E2f5 DKO retinae that had cellular features of immature cells as described previously (Fig. S4) [9].

When combined, our analyses of these 9 strains of mice (Fig. 1 and 2) suggest that E2f1 is unique in its ability to rescue both retinoblastoma formation in the 7D model and the retinal development phenotype in the Rb cKO model. However, the rescue was not complete. The cells in the ONL with features of immature cells found in the 7D retinae and the Rb cKO retinae were also present in the E2f1 DKO and E2f1 TKO retinae (Fig. S4). These immature cells can be further subdivided into Type I and Type II (Fig. S4). Type I cells are characterized by cell somata of size and location similar to normal rods. In some cases, Type I cells appeared to contain vestiges of spherule elements. Thus, Type I could represent arrest of rod development at later stages or incomplete arrest. Type II cells remain in the ONL or migrate proximately along with some “normal” rod somata, but have a very large, pleomorphic nucleus and uniformly dispersed chromatin (with a unique nuclear inclusion body), as well as extensive processes somewhat vertically arranged. This could potentially represent arrest of rod development at early stages or complete arrest. Both Type I and II cells were found in the E2f1 DKO and E2f1 TKO retinae. Overall, these cells were rare and our analysis shows that E2f1 is able to largely rescue both the tumor and developmental phenotype, E2f3 is able to rescue the tumor phenotype but not the developmental phenotype and E2f5 can rescue neither.

### Pax6 Inactivation Inhibits Tumorigenesis in Rb-deficient Retina

**Figure 3:**
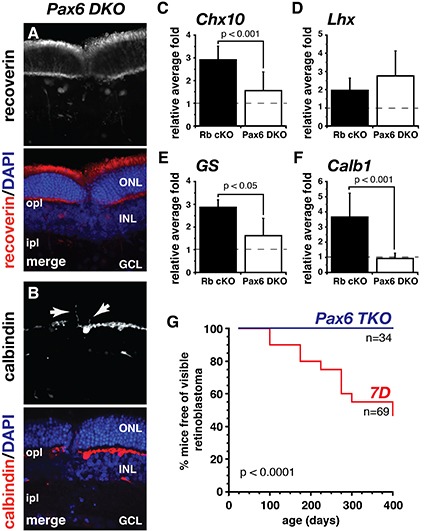
Pax6 rescues tumorigenesis but not retinal development on a retinoblastoma-sensitized background **(A)** Recoverin immunostaining (red) labeled photoreceptors in the ONL and DAPI nuclear counterstain (blue) showed that the ONL, containing photoreceptors, is thinner in patches of *Chx10-Cre;Rb^lox/lox^;Pax6^lox/lox^* (*Pax6 DKO*) retinae. **(B)** Calbindin immunostaining (red) labeled a subset of amacrine and horizontal cell bodies in the INL, as well as their respective synaptic processes in the IPL and OPL, respectively. In *Pax6 DKO* retina, calbindin immunopositive horizontal cell processes extended into the ONL (arrows). Nuclei were counterstained with DAPI (blue). **(C-F)** Real-time RT-PCR analysis showed several genes overexpressed in *Rb cKO* retinae are restored back to wild type levels in *Pax6 DKO*. All data are normalized to their wild type or control littermates (n=5). **(G)** Kaplan–Meier curve showing the time to first observation of externally visible retinoblastoma. These times were markedly increased in *Chx10-Cre;Rb^lox/lox^; p107^−/−^;Pax6^lox/lox^* (*Pax6* TKO) relative to *Chx10-Cre;Rb^lox/lox^;p107^−/−^ (7D*) littermates. ONL, outer nuclear layer; INL, inner nuclear layer; OPL, outer plexiform layer; GCL, ganglion cell layer; ipl, inner plexiform layer; opl, outer plexiform layer.

Pax6 and Chx10 repress rod photoreceptor development, and these genes are upregulated in Rb-deficient retinal cells in the ONL [17–19]. To determine if the rescue of rod differentiation observed by eliminating E2f1 from the Rb-deficient retinae is acting through the control of the active transcription of Pax6 and Chx10, affecting rod photoreceptors cell fate, we analyzed gene expression using qPCR at P21. We found that *Pax6* and *Chx10* gene expressions are restored back to wild-type (wt) levels both in E2f1 DKO and E2f3 DKO retinae (Fig. 2K and L) even though E2f3 DKO retinae still have a defect in rod differentiation. These data suggest that Pax6 and/or Chx10 are not sufficient to cause the retinal differentiation phenotype in the Rb-deficient retinae. To confirm this observation, we generated *Chx10-Cre*;*Rb^lox/lox^*; *Pax6^lox/lox^* (Pax6 DKO) mice. We harvested retinae from Pax6 DKO mice at P21 and performed immunohistochemical analysis of molecular markers for the different retinal cell types. We observed no significant differences in the expression pattern of these markers in the retina, when compared to Rb cKO mice, with thinning of the outer nuclear layer and ectopic horizontal cell processes still being present (Fig. 3A, B). However, in Pax6 conditional knockout retina (Pax6 cKO), retinal ganglion cells, rod and cone photoreceptors, bipolar cells, horizontal cells, and Müller glia cells fail to develop [20]. This precludes our ability to distinguish the retinal phenotype characteristic from Pax6 cKO from that of Rb cKO. Despite this limitation, loss of Pax6 rescues some of the aberrant gene expression observed in Rb-deficient retinae, including *Chx10*, *GS* and *Calb1* (Fig. 3C-F).

E2f1 TKO and E2f3 TKO retinae fail to form tumors and as mentioned above, the Pax6 expression levels are restored to wild type levels in these E2f1 and E2f3 DKO retinae. To determine whether Pax6 upregulation in the 7D retina has an effect in retinoblastoma tumorigenesis, we generated *Chx10-Cre;Rb^Lox/Lox^; −107^−/−;^;Pax6^Lox/Lox^* (Pax6 TKO) mice and aged them for 400 days. Surprisingly, Pax6 TKO mice failed to develop visible tumors up to 400 days old (Fig. 3G). We speculate that among the retinal cell types that fail to develop due to loss of Pax6 is the retinoblastoma cell of origin and therefore, no tumors are formed. TEM analysis of Pax6 TKO mice support the hypothesis that the retinal cell of origin fails to develop in the absence of Pax6 as Pax6 TKO retinae show no evidence of the persistent progenitors observed in aE2f TKO mice (data not shown).

### Identification and Characterization of Rb Target Genes in Retinal Development

**Figure 4:**
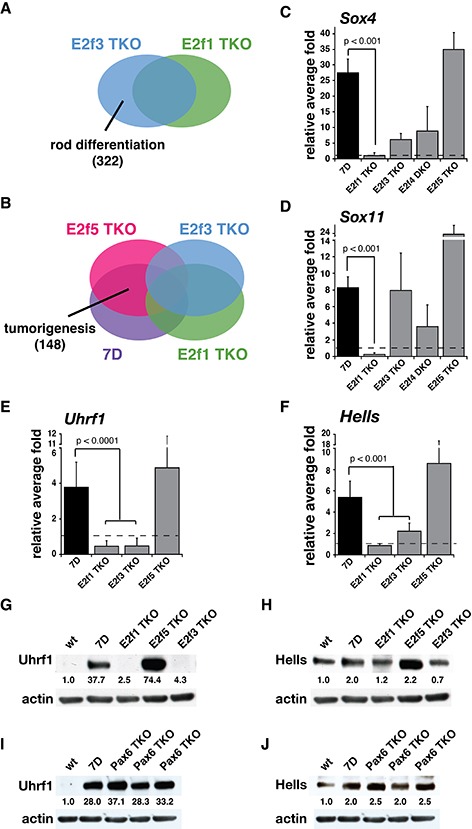
Identification and characterization of Rb target genes in retinal development and retinoblastoma **(A-B)** Venn diagrams of the gene expression array comparisons used to identify potential Rb target genes involved in **(A)** rod differentiation and **(B)** tumorigenesis. Number of genes identified in parenthesis. **(C-F)** Real-time RT-PCR analysis shows increased *Sox4* **(C)** and *Sox11* **(D)** mRNA levels in P21 retinae from *Chx10-Cre;Rb^lox/lox^;p107^−/−^* (*7D)*, *Chx10-Cre;Rb^lox/lox^;p107^−/−^;E2f3^lox/lox^* (*E2f3* TKO), *Chx10-Cre;Rb^lox/lox^; E2f4^−/−^* (*E2f4* DKO) and *Chx10-Cre;Rb^lox/lox^;p107^−/−^;E2f5^lox/lox^* (*E2f5 TKO*) but not *Chx10-Cre;Rb^lox/lox^;p107^−/−^;E2f1^−/−^* (*E2f1 TKO*) compared to wt retinae. It also shows increased *Uhrf1* **(E)** and *Hells* **(F)** mRNA levels in P21 retinae from *7D* and *E2f5 TKO* but nor *E2f1 TKO* and *E2f3* TKO compared to wt retinae. Each bar is the mean and standard deviation of 5 biological replicates ran in duplicate. **(G-J)** Western blot analysis from P21 mouse retinae show increased Uhrf1 **(G)** and Hells **(H)** protein levels in *7D* and *E2f5 TKO* retinae but not *E2f1 TKO* and *E2f3 TKO* retinae compared to wt. **(I)** Uhrf1 and **(J)** Hells protein levels are also overexpressed in *Chx10-Cre;Rb^lox/lox^;p107^−/−^;Pax6^lox/lox^* (*Pax6* TKO) retinae at P21 when compared to wt. Data were normalized to actin.

Since our different E2f models provide distinct phenotypes, we performed differential gene expression array analysis using P21 retinae from a subset of the 9 mouse strains described here (Supplemental Information). Gene expression arrays from five mice from each strain were compared against wt retinae and a 2-fold change cutoff was used for selection of genes differentially expressed. In order to identify genes that may contribute to retinal development we compared the gene expression array data for the one strain that rescued retinal development (E2f1 TKO) to another one that have retinal defects (E2f3 TKO). We identified 322 genes that were differentially expressed between E2f3 TKO and E2f1 TKO (Fig. 4A, Table S1). Of these, we identified 17 genes with known functions in retinal development (Fig. S5). Of interest among these 17 genes, *Sox4* and *Sox11* are two genes that play pivotal roles in retinal development, in particular photoreceptor genesis [21, 22]. We were able to validate the upregulation of these genes at the mRNA level (Fig. 4C, D) in the E2f mouse models with defects in photoreceptor genesis (Rb cKO, E2f3 TKO, E2F4 DKO, and E2f5 TKO), but not in the model that rescues proper retinal development (E2f1 TKO). We hypothesize that early expression of Sox4 or Sox11 may cause precocious photoreceptor genesis and subsequent degeneration. Sox4 and Sox11 are not direct E2f targets, but are known to associate with Rb/E2f to control cell cycle [23–25].

### Identification and Characterization of Rb Target Genes and miRNAs in Retinoblastoma

In order to identify genes in addition to Pax6 that may contribute to tumorigenesis, we compared the gene expression array data for two strains that do develop tumors (7D and E2f5 TKO) to two strains that did not develop tumors (E2f1 and E2f3 TKO). We identified 148 genes that were differentially expressed in 7D and E2f5 TKO retinae but were similar to normal P21 retinae in E2f1 TKO and E2f3 TKO retinae (Fig. 4B, Table S2). Gene ontology functional analysis of these 148 genes showed a significant enrichment for genes involved in cell cycle and proliferation, cell death and eye and lens development (Table S2 and Fig. S6). Of these 148 genes, 40 genes were previously reported as epigenetically deregulated in mouse retinoblastoma [26] and 25 genes also deregulated in human retinoblastoma [5] (Table S2).

Differential expression levels for miRNAs were also evaluated given that previous studies have shown a role of miRNAs in retinoblastoma formation [27, 28]. Table 1 shows the list of miRNAs that are deregulated in the 7D retinoblastoma model as well as the direction of the expression changes in E2f1 TKO and E2f3 TKO at P21. As seen in Table 1, miRNA deregulation is likely not involved in the retinal degeneration phenotype characteristic of Rb loss as both E2f1 TKO and E2f3 TKO retinae show similar patterns of miRNA expression. Interestingly, both aE2f TKOs were able to restore wt miRNA levels for the majority of miRNAs deregulated in 7D mice, including the miR17~92 cluster associated with retinoblastoma tumorigenesis [27, 28].

**Table 1:** Rb-mediated miRNA deregulation at P21 is rescued by loss of E2F1 and E2F3

		Expression compared to wt retina	
miRNA	Rb/p107	Rb/p107/E2F1	Rb/p107/E2F3
mir-15a	down	no change	no change
mir-17	up	no change	no change
mir-18a	up	no change	no change
mir-20a	up	no change	no change
mir-20b	up	no change	no change
mir-22	down	no change	no change
mir-26b	down	no change	no change
mir-29cstar	down	no change	no change
mir-30bstar	down	no change	no change
mir-34b-3p	up	no change	no change
mir-34c	up	no change	no change
mir-93	up	no change	no change
mir-96	down	no change	no change
mir-106a	up	no change	no change
mir-124a(TM1182)	down	no change	no change
mir-124star	down	down	down
mir-142-3p	up	up	up
mir-143	down	no change	no change
mir-145	down	no change	no change
mir-146a	up	no change	no change
mir-146b	up	no change	no change
mir-182	down	no change	no change
mir-183	down	down	down
mir-183star	down	no change	no change
mir-335star	down	no change	no change
mir-378(TM2243)	down	no change	no change
mir-378(TM567)	down	no change	no change
mir-449b(TM1667)	up	no change	no change
mir-672	up	no change	no change
mir-872star	down	no change	no change

### Epigenetic Regulators Contribute to Retinoblastoma Tumorigenesis

Previous studies have demonstrated that retinoblastoma’s tumor progression is mediated through an epigenetic process [5, 26]. Therefore, we looked for genes involved in chromatin regulation within the retinal development (Table S1) and tumorigenesis (Table S2) candidate tables. Since these lists contain genes deregulated in retinae by P21, they could play a role in the early events that lead to chromatin remodeling and promote tumorigenesis in retinoblastoma. Within these, we identified 11 genes involved in chromatin organization (Fig. S5 and S6). Among those, *Uhrf1* and *Hells* were two candidate genes, which have been previously reported to participate in tumorigenesis [29, 30]. We were able to validate the upregulation of these genes both at the mRNA and protein level (Fig. 4E-H) in the E2f mouse models that develop retinoblastoma tumor, but not in those that rescue the tumorigenic phenotype (E2f1 TKO and E2f3 TKO). Intriguingly, Pax6 TKO retinae express high levels of both Uhrf1 and Hells protein even though they do not form tumors (Fig. 4I, J). If our hypothesis of the tumor cell of origin not being present in the Pax6 TKO retinae, this results suggested that upregulation of Uhrf1 and Hells is only tumorigenic in the context of the tumor cell of origin.

**Figure 5:**
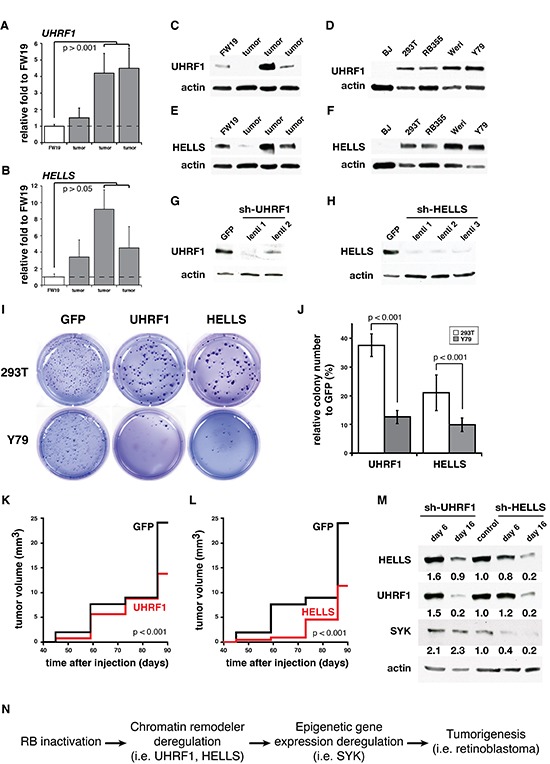
UHRF1 and HELLS are required for human retinoblastoma survival **(A-B)** Real-time RT-PCR analysis shows several human retinoblastoma tumors with increased gene expression for **(A)** UHRF1 and **(B)** HELLS compared to fetal week 19 normal retina (FW19). All data are normalized to FW19. Each bar is the mean and standard deviation of triplicate experiments. **(C, E)** Western blot analysis from FW19 and human retinoblastoma tumors show increased **(C)** UHRF1 and **(E)** HELLS protein expression in a subset of human tumors. **(D, F)** Western blot analysis from normal diploid fibroblasts (BJ), human embryonic kidney 293 cells (293T), and human retinoblastoma cell lines (RB355, Weri and Y79) show increased **(D)** UHRF1 and **(F)** HELLS protein expression in retinoblastoma cell lines. **(G-H)** Western blot of 293T cells infected with the lentiviral vectors encoding GFP as a negative control or shRNA against **(G)** Uhrf1 or **(H)** Hells. All Western blot data were normalized to actin. **(I)** Representative image from colony forming assay plates with 293T or Y79 cells infected with lenti-UHRF1 or lenti-HELLS compared to control GFP. **(J)** Histogram of the proportion of the number of colonies formed in 293T and Y79 cells following infection with the control GFP or lenti-UHRF1 or lenti-HELLS. Each bar is the mean and standard deviation of duplicate experiments. **(K-L)** Tumor burden curve show that infection with **(K)** lenti-UHRF1 or **(L)** lenti-HELLS delayed the progression of the tumors compared to control GFP (n=8). **(M)** Western blot analysis from Y79 cells infected with lenti-UHRF1 or lenti-HELLS show that knock-down of HELLS but not UHRF1 decreases SYK protein levels. Densitometry quantification normalized to control is shown below each blot. **(N)** Rb-mediated epigenetic deregulation hypothesis.

Interestingly, researching the data from our previous integrative analysis of human retinoblastoma, we found that the human orthologs, UHRF1 and HELLS, were both epigenetically upregulated in human retinoblastoma [5]. Indeed, qPCR analysis of primary human retinoblastoma tissue showed that UHRF1 and HELLS are significantly upregulated in some retinoblastomas (Fig. 5A and B). At the protein level, we identified overexpression of UHRF1 and HELLS in a subset of the human retinoblastoma tumors and in all the human retinoblastoma cell lines analyzed (RB355, Weri, and Y79) when compared to normal fetal retina (FW19) and normal human fibroblasts (BJ) (Fig. 5C-F).

To determine whether UHRF1 or HELLS expression are required for retinoblastoma’s growth, survival, or both, we acquired lentiviral vectors encoding shRNAs to UHRF1 (lenti-UHRF1) and HELLS (lenti-HELLS). All of these lentiviruses efficiently knocked down UHRF1 and HELLS in the 293T cell line and retinoblastoma cell lines (Fig. 5G-H). Next, we infected retinoblastoma cell lines (Y79, RB355, and Weri) with a mixture of lenti-UHRF1 or lenti-HELLS and measured their ability to form colonies in a soft agar as a measure growth and survival after 21 days (Fig. 5I-J and data not shown). We used a lentiviral vector expressing GFP (lenti-GFP) and a non-tumorigenic cell line that expresses both UHRF1 and HELLS (293T) as controls. UHRF1 and HELLS knockdown in both control and retinoblastoma cell lines led to a significant reduction in colonies formed compared to the lenti-GFP controls. However, the impact of UHRF1 and HELLS knockdown was significantly more pronounced in retinoblastoma cell lines than in 293T controls and while in 293T the decreased number of colonies led to larger colonies, retinoblastoma cell colonies were fewer in number and unaltered in size (Fig. 5I-J and data not shown).

We used our SJRB001X orthotopic xenograft model [31] to test the efficacy of lenti-UHRF1 and lenti-HELLS in vivo by infecting cells prior to their injection into the eye and monitored the size of the tumors formed using MRI (Fig. 5K-L). Compared to the lenti-GFP infected tumors, lenti-HELLS infected tumors showed both a delay in the growth of the tumor as well as a reduced tumor burden by the end of the study (Fig. 5L). On the other hand, while lenti-UHRF1 infected tumors grew at the same rate as lenti-GFP, their final tumor size was significantly smaller than the control mice (Fig. 5K). We also assessed whether the epigenetic upregulation of SYK was mediated by either UHRF1 or HELLS. We found that knocking down HELLS but not UHRF1 significantly reduced the SYK levels in Y79 cells (Figure 5M). This suggested that HELLS and UHRF1 depletion induce tumor growth inhibition through independent mechanisms. Interestingly, knock-down of HELLS also affects the protein levels of UHRF1 similar to lenti-UHRF1 inhibition, but the reverse is not completely true. Further studies are warranted. Taken together, these data suggest that targeting HELLS may be an effective treatment strategy in retinoblastoma or an alternative approach to identifying novel therapeutic targets.

## DISCUSSION

Little is known in retina about the mechanism(s) through which Rb regulates developmental events in coordination with cell cycle regulation. One theory is based on the fact that Rb can bind to more than 150 proteins, many of which are cell type–specific transcription factors [1, 32]. Therefore, depending on the cellular context, Rb and cell type–specific transcription factors may work together to regulate specific developmental programs, while Rb executes cell cycle exit through its canonical mechanism. In addition, Rb, p107, and p130 act through different E2f family members to regulate transcription. Also, not only do different E2fs regulate distinct sets of genes, but also they act through different mechanisms that may contribute to the complexity of the coordinated regulation of cell cycle exit and cell fate specification during cellular differentiation. In this study, we demonstrate that the retinal differentiation defects and tumorigenesis that follow after the loss of the Rb family occur as two independent events that rely on distinct E2f family members. We also identified UHRF1 and HELLS as two chromatin remodeler proteins that may play key roles in the Rb phenotype, including the epigenetic rearrangements that promote retinal tumorigenesis (Figure 5N) [5, 26].

### Activator E2fs Role in Retinal Development and Retinoblastoma

Epigenetic processes can act as a major cancer driver in the absence of other genetic lesions in cancers like retinoblastoma [5]. Here, we tried to elucidate the mechanism by which Rb inactivation leads to the epigenetic changes that contributes to tumorigenesis. We found that inactivation of either *E2f1* or *E2f3* can suppress tumor development in the retina. These results are in line with other studies, which have reported that the loss of transcriptional control of activator E2Fs (E2f1 and E2f3 in particular) is responsible for tumorigenesis following inactivation of the Rb gene [3, 12, 33–35]. Yet, the previous study in the retina identified E2f1, but not E2f3, as a potential mediator of the Rb-mediated tumorigenesis [12]. This difference could be explained by the distinct Cre-loxP systems (Chx10-Cre compared to alpha-Cre) used to mediate recombination of the conditional alleles in these studies. However, only E2f1 abrogation was able to restore proper retinogenesis. For years, failure of photoreceptors to terminally differentiate in Rb-deficient retinae was used to support the idea that photoreceptors are the cell-of-origin of retinoblastoma. Our data suggests that photoreceptor differentiation and tumorigenesis are not linked because we could suppress tumorigenesis both in a background that restored rod differentiation (E2f1 TKO) and one that did not (E2f3 TKO).

Also, our results support a role of E2f1 and E2f3 in retinoblastoma formation through deregulation of miRNA expression. The miR-17~92 cluster, which is highly expressed both in murine and human retinoblastoma, synergizes with the loss of Rb family members to promote retinoblastoma [28]. Here, we show that miR-17~92 is upregulated early in the tumorigenic process (by post-natal day 21 in the mouse). We also found that the up-regulation of miR-17~92 that follows deletion of Rb family members is likely mediated through the deregulation of E2f1 or E2f3 as loss of either aE2f restores wild-type miRNA levels of this and other for miRNAs deregulated in 7D mice. In addition, our data further supports the absence of a role of miRNA in the retinal development defects that follow Rb-loss since ablation of either E2f1 or E2f3 rescue the aberrant miRNA expression observed in Rb/p107-deficient retinae, but only E2f1 abrogation rescues retinal development. Previous studies have reported that Dicer1 inactivation in normal and Rb/p107-deficient mice did not affect retinogenesis [27] and that miR-17~92 was dispensable for mouse retinal development [28].

### Persistent Progenitors: Remnants of the Retinoblastoma Cell of Origin?

A puzzling finding from the TEM analyses of the different E2f TKO mice was the identification of a distinct cell type with progenitor-like features, which we termed “persistent progenitors”. Many of their features, including spherule vestiges, point at the possibility that these cells could potentially represent an early stage arrest of rod photoreceptor development. This hypothesis is supported by our immunohistochemistry staining observations that the ONL shows only rod markers in these retinae. Interestingly, persistent progenitors were not present in Pax6 TKO retinae. We have hypothesized that the absence tumor formation in Pax6 TKO is due to loss of Pax6 preventing the formation of the retinal subtype that gives rise to retinoblastoma. If true, elucidating the origin of the persistent progenitors warrants further studies as they may represent the cell of origin of retinoblastoma. It is possible that among the retinal cell types that fail to develop due to loss of Pax6 is the retinoblastoma cell of origin and therefore, no tumors are formed. If the persistent progenitors observed in aE2f TKO mice are remnants of the retinoblastoma cell of origin, results from TEM analysis of Pax6 TKO mice support the hypothesis that the retinal cell of origin fails to develop in the absence of Pax6 as Pax6 TKO retinae show no evidence of persistent progenitors (data not shown).

### Retinoblastoma Epigenetic Tumor Progression: HELLS and UHRF1

The generation of novel GEMMs used in combination with differential gene expression and miRNA analyses has provided us with tools to identify potential transcriptional targets of Rb in the developing retina. These may provide insight to the mechanism(s) of Rb-mediated epigenetic regulation that leads to tumorigenesis. In particular, we have identified *Uhrf1* and *Hells* as two genes encoding proteins involved in chromatin remodeling that may be involved in the epigenetic changes observed in retinoblastoma and required for tumor survival.

Uhrf1 can recruit histone deacetylases (HDAC) and DNA-methyltransferase 1 (Dnmt1) to specific DNA sequences to regulate gene expression and maintain the chromatin structure (reviewed in [30]). Uhrf1 is also known to play a role in G1/S transition by regulating topoisomerase II alpha (Top2a) and Rb gene expression [30]. Indeed, consistent with an upregulation of Uhrf1 we also observed upregulation of Top2a (data not shown). Recently, UHRF1 has been classified as an oncogene that is highly expressed in many cancers [30]. Interestingly, our previous analysis of the epigenetic landscape of human retinoblastoma identified UHRF1 as a gene epigenetically deregulated in retinoblastoma [5]. Also consistent with our findings in this study, a recent report identified UHRF1 as a target of E2F1 [36–38]. Another chromatin remodeling-associated protein we found overexpressed in retinoblastoma is Hells. Hells is a Swi/Snf-related matrix-associated actin-dependent regulator of chromatin. Hells remodels chromatin to render DNA accessible to DNA methyltransferase enzymes Dnmt3a or Dnmt3b, but not Dnmt1, and therefore supports de novo DNA methylation and stable gene silencing [39]. Like UHRF1, HELLS was also epigenetically upregulated in human retinoblastoma [5]. Furthermore, we identified that upregulation of HELLS following RB1 inactivation is linked to the epigenetic upregulation of spleen tyrosine kinase (SYK, Figure 5M), a protein previously described to be key for human retinoblastoma survival [5]. While HELLS has not been identified as a direct E2F target, HELLS protein has been shown to interact with E2F3 at several E2F target genes that control cell cycle entry [29]. Similar to what we observed for retinoblastoma, depletion of HELLS in prostate cancer cell lines impaired growth, suggesting that HELLS may contribute to the malignant progression of tumors [29].

Together, our data implies that loss of the Rb family alters the two types of DNA methylation pathways known to date: methylation maintenance at the replication fork through deregulation of UHRF1 and de novo methylation through deregulation of HELLS. This could have important implications for cancers other than retinoblastomas as the mechanisms involved in this epigenetic landscape rearrangement may be are conserved in other tumors with RB1 inactivation and/or may serve as a way to start deciphering why some, but not all, cells become tumors upon RB1 inactivation.

## MATERIALS AND METHODS

### Xenografts, Mouse Models of Retinoblastoma, and Cell Lines

The orthotopic xenograft (SJRB001X) has been described previously [5, 13]. Two additional orthotopic xenografts were also used in this study (SJRB002X and SJRB004X), and the primary tumors that gave rise to these xenografts have also been described previously [13, 26]. Severe combined immunodeficiency mice (SCID) were obtained from Jackson laboratories (B6.CB17-Prkdc<scid>SzJ). Fresh human fetal retinal samples were obtained from ABR, Inc.

The *p107*-knockout mice were obtained from Dr. Tyler Jacks (Massachusetts Institute of Technology); *Chx10-Cre* mice were obtained from Dr. Connie Cepko (Harvard Medical School); *Rb^Lox/Lox^* mice were obtained from the Mouse Models of Human Cancer Consortium at the National Cancer Institute; *E2f1^−/−^* and *E2f3^Lox/Lox^* mice were obtained from Dr. Gustavo Leone (The Ohio State University); *E2f4^−/−^* and *E2f5^Lox/Lox^* mice were obtained from Dr. Joseph Nevins (Duke University); *Pax6 ^Lox/Lox^* mice were obtained from Ruth Ashery-Padan (Tel Aviv University). Mice were monitored weekly for signs of retinoblastoma and anterior chamber invasion. Moribund status was defined as the point when tumor cells invaded the anterior chamber and intraocular pressure increased to the point of imminent ocular rupture. The St. Jude Laboratory Animal Care and Use Committee approved all animal procedures.

Retinoblastoma cell lines Y79, Weri1, and RB355 were cultured in RPMI medium (Lonza RPMI-1640) supplemented with 10% fetal bovine serum (Equitech Bio.), penicillin, streptomycin, and glutamate (Gibco). Cells were passaged every 3 to 4 days or when they reached 70% to 80% confluence. At the time of passage, cells were split to 20% confluence. BJ cells were grown in EMEM supplemented with 10% FBS; 293T cells were grown in DMEM supplemented with 10% FBS.

### Gene Expression Arrays

Gene expression arrays were analyzed as described previously [13].

### Real-Time RT-PCR

Real-time RT-PCR experiments were performed using the Realplex^2^ Mastercycler (Eppendorf). Primers were designed using Primer Express® software (Applied Biosystems, Supplemental Information). RNA was prepared using Trizol, and cDNA was synthesized using the Superscript system (Invitrogen, Carlsbad, CA). Samples were analyzed in duplicate and normalized to 18S and Gpi1 expression levels. We also custom designed Taqman 384-well expression (Applied Biosystems): *Dtl* (Mm00712787_m1), *Hells* (Mm00468580_m1), *Gfap* (Mm01253033_m1), *Mcm2* (Mm00484804_m1), *Rrm2* (Mm00485881_g1), *Tcf19* (Mm00508531_m1), *Top2a* (Mm00495703_m1), *Uhrf1* (Mm00477873_g1). 500ng of sample RNA was used for cDNA synthesis (High Capacity RNA-to-cDNA kit; Applied Biosystems #4387406).

### Immunohistochemistry

Retinas were isolated in PBS and fixed for 1 hr in 4% (w/v) paraformaldehyde. Whole retinas were embedded in 4% (w/v) agarose in PBS and cut into 50-μm sections using a vibratome. Retinal sections were blocked in 5% (v/v) normal goat serum, 0.5% (v/v) Triton X-100 in PBS for 3 hr at room temperature, and incubated in primary antibody in the same block solution o/n. Mouse anti-calbindin antibody (C-9848, Sigma) was used at 1:100, and rabbit anti-recoverin antibody (AB5585, Millipore) was used at 1:5000. Donkey anti-mouse biotin antibody (Vector Labs) at 1:500 and Goat anti-rabbit biotin antibody (Vector Labs) were incubated for 1 hr, followed by 30 min incubation in VECTASTAIN ABC Kit (Vector Labs) and 10 min in tyramide Cy3 1:100 in amplification buffer (Perkin Elmer). Nuclei were stained with DAPI. Images were acquired with a Zeiss LSM700 confocal microscope.

### Western Blotting

Samples were lysed for 30 min on ice in RIPA buffer (790 mg Tris Base, 880 mg NaCl, 5 mL of 20% NP40, 2.5 mL of 10% deoxycholate, 0.2 mL of 500 mM EDTA, up to100 mL, pH 7.4) containing protease inhibitor cocktail (11836153001, Roche Diagnostics, IN, USA). Lysates were cleared by centrifugation at 15000 RPM at 4° for 30 min. Protein concentration from cell lysate was measured by using a BCA protein assay kit (232225, Pierce). 20 ng of total protein were resolved in 4-15% SDS-PAGE gel.

(Bio-Rad) and transferred to PDVF membrane (Millipore). Non-specific binding was prevented blocking the membrane with 3% non-fat dry milk in TBS-0.25% Tween (TBS-T) for 1 hr at RT. The membranes were incubated at 4°C o/n in primary antibody: 1:500 anti-SYK (2712, Cell Signaling), 1:1000 anti-human Uhrf1 (ab57083, Abcam), 1:500 anti-mouse Uhrf1 (sc-373750, Santa Cruz Biotechnology), 1:1000 anti-Hells (sc-28202, Santa Cruz Biotechnology), 1:5000 anti-actin (A1978, Sigma). Membranes were rinsed 3 times for 15 min with TBS-T on shaker and incubated with anti-mouse or anti-rabbit IgG conjugated to horseradish peroxidase (Dako) 1:5000 for 1 hr at RT. Following a 3 washes with TBS-T for 15 min, protein signals were detected using the SuperSignal West Dura Extended Duration Substrate (34075, Pierce).

### Lentiviral Vector Preparation and Infection

GIPZ shRNA viral particles specific for human UHRF1 and HELLS were obtained from Thermo Fisher Scientific (Pittsburgh, PA, USA). Lentivirus production was performed by co-transfecting the viral plasmid and 3 packaging plasmids into HEK293T cells with polyethylenimine. Supernatants containing the lentiviral particles were harvested at 48 hours post-transfection, filtered, and concentrated by ultracentrifugation at 24,000 rpm for 90 min at 4°C. Infections were performed exposing the cells to concentrated viral supernatant for 4 hrs.

### Colony Formation Assay

Lentivirus infected cells were centrifuged and resuspended in pre-warmed (37°C) 0.7% agarose solution in medium and plated at low-density into 35-mm cell culture plates pre-coated with 0.8% agar in the appropriate cell culture media. Fresh medium was added every 3–4 days. When sufficient colonies were visible, typically after 14–21 days, cells were washed once in PBS before fixing in ice-cold methanol for 30 min while shaking. Methanol was aspirated and Giemsa stain added at a dilution of 1:20 overnight while shaking. The following day cells were rinsed in distilled water and air-dried.

### Magnetic Resonance Imaging for Tumor Volume Determination

Magnetic Resonance Imaging (MRI) was performed using a 7-Tesla Bruker Clinscan animal MRI scanner (Bruker BioSpin MRI GmbG, Germany) equipped with Bruker 12s gradient (BGA12S) and a four channel phase-array surface coil placed on the mouse’s head. Mice were anesthetized using isoflurane (2–3% in O2) for the duration of the data acquisition. 3D Magnetization Prepared Rapid Gradient Echo (MP-RAGE) protocol (TR 2500 ms; TE 2.5 ms; TI 1050 ms) was used to produce T1 weighed images (coronal) using a matrix of 256 × 146 and FOV of 30 × 20.6 mm. The slice thickness for the T1 weighted image was 0.5 mm. The initial images were read on a Siemens workstation using Syngo MR B15 software (Siemens, Erlangen, Germany) and reviewed with MRIcro (Version 1.4) Software.

## SUPPLEMENTARY MATERIALS AND METHODS


