# Supplementary material for: Chromatin remodelers HELLS and UHRF1 mediate the epigenetic deregulation of genes that drive retinoblastoma tumor progression
Source: Oncotarget. 2014 Oct 13;5(20):9594–608. doi: 10.18632/oncotarget.2468 (PMC4259422; doi:10.18632/oncotarget.2468)
Supplement: Supplementary file 1 [file oncotarget-05-9594-s001.pdf]

# SUPPLEMENTARY MATERIALS AND METHODS

## Transmission electron microscopy

All procedures for TEM have been described previously (Johnson et al. 2006). Animals were anesthetized with Avertin until a loss of deep tendon reflexes. Transcardial perfusion was performed with carboxygenated Ames Medium supplemented with 40 mM glucose to clear the vasculature, followed by perfusion with Sorenson's phosphate buffer pH 7.2 with 2% EM grade paraformaldehyde and 1% EM grade glutaraldehyde. Eyes were then harvested, a slit was made in the cornea to aid in diffusion, and the tissue was placed in 3% glutaraldehyde in Sorenson's phosphate buffer overnight. Tissue was washed with 0.2 M cacodylate buffer in 5% sucrose, post-fixed in 1% $OsO_4$, embedded, sectioned, and viewed by transmission electron microscopy (TEM).

## Real-time Primers and Probes

| Gene | Forward | Reverse | Probe |
|---|---|---|---|
| mArr3 | TGACTTGGATGTGATTGGTCTGA | GGAGCCACTTGCTTGGTTTG | ATTTCGCAAAGATCTCTATG |
| mBrn3b | CCGGTTTCGCAGTGAAGAA | GCTCTGCTACAACTTGACAAATTCC | AACCAAAACCACAAACAACACAACACGAAA |
| mCalb1 | CACATGTAACTCTGTTTCGTGTATCCTT | TCACAATAAAGAATCCAGGCAATTAA | AGTGTGTCCTCTGCTGGTTATTGGTGACAA |
| mChx10 | TGAGGCAAGGCCCATGTC | CGGGAGTATGTCCAGGATGTCT | AGGCCCGCAATTACCCCCACG |
| mCRALBP | CTGTCCAGGGTGGAGGTCAT | CCCCAGCACCAAGGATCAC | TCCCTTCCTCTCCACCCATCTTGGA |
| mCrx | TCTGTGTGTTACAGACATGACCACTAA | CATCAAGCTTCTTTTGCATTTTGT | CTGAGCTGGGATGCTGTGGCTTGTATAAA |
| mGAPDH | CTCCACTCACGGCAAATTCA | CGCTCCTGGAAGATGGTGAT | AAGGCCGAGAATGGGAAGCTTGTCATC |
| mGFAP | ATCGAGAAGGTCCGCTTCCT | GGCTCGAAGCTGGTTCAGTT | AGCAAAACAAGGCGCTGGCAGC |
| mGS | GGTGCCAAGTTTGAGTGATGAG | ACTTTCCCGGTACTGCATCCT | CTTGGCTTAGAAGTGAGGCTCCCTTGAGG |
| mGnat1 | TGACCAATAATGAAGCCATGCT | TCTTTGCCCATGGTTGATCA | TGGCTCAGAGCAGAGTCCAACCCCTA |
| mGPI-1 | TCCGTGTCCCTTCTCACCAT | GGCAGTTCCAGACCAGCTTCT | CTCCCTGCCCAGAGCGCACC |
| mGrm6 | TTCCTGAAGACAGAGACTAAATTGACA | GGCAAGCCCTGACTTTACCA | TCTGGGCGGGCATTCAAACCC |
| mHes1 | AGAGCCTCAGGCCACTGCTA | TTCGTTTTTAGTGTCCGTCAGAAG | CCGTAAAGTCCCTAGCCCACCTCT |
| mNes1 | CAACGCCCCCTTTCTTCTG | GAGCAGTCTCGTGGGAAAGC | CACCTGCTGGAAGAGGCTTGGGC |
| mNr2e3 | AGGCTGGAAGTTGAACAAAAGC | TCCAGTCTCCCTCCTTTCCC | AGGCACGTTTATCCTGTGTTCAAGACCAGA |
| mNrl | AACTTCTGAGCATCGTGGCA | TGAAGAGTCGTGACCTGCAAA | CTGGTCCTTTGCTGGAGATGAGTTCCCT |
| mPax6 | CACCGCCCTCACCAACAC | AGGTTGTTTGCCATGGTGAAG | AGTGCTTTGCCACCCATGCCCA |
| mPrkca | CGCGAGGACAGCCTGTCT | AGAACCCTTCAAAATCAGATTGGT | CCACCAGATCAGCTGGTCATTGCTAACATA |
| mProx1 | TTAGAACAGGCTCGTGGTGAGA | TGAAGCAAAGTAAATAGCAACTAGTGACA | TGAGCTTGGCCGTGTGGACTTAGAGG |
| mRcvrn | GCAGCTTCGATGCCAACAG | TCATGTGCAGAGCAATCACGTA | TACGCTGGACTTCAAGGA |
| mSyt1 | CCACTGCCCTCCAAATCTACTC | ACAACGGTGTGATACAATACATTTCA | TCTTTTAAGCAATATGATGTGTAGATAGAGC |

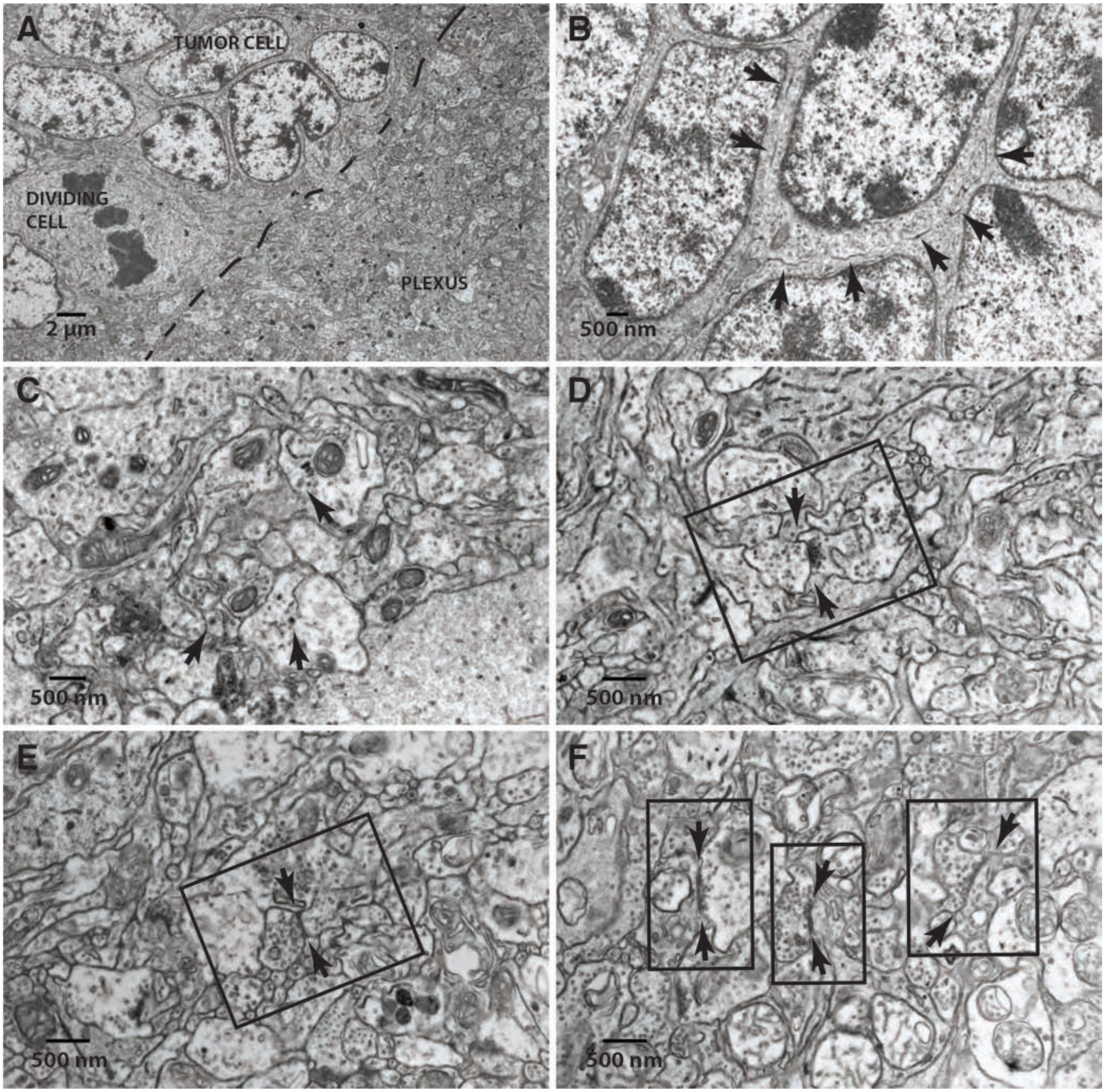


**Supplementary Figure S1: Transmission Electron Microscopy from E2f5 TKO Tumors.** **(A-E)** Transmission electron microscopy (TEM) from a *Chx10-Cre;Rb$^{lox/lox}$;p107$^{-/-}$;E2f5$^{lox/lox}$* (*E2f5 TKO*) tumor show that these tumors have all of the differentiation features of 7D and human retinoblastomas with **(A)** dividing cells, tightly packed tumor cells with areas of plexus, **(B)** cell to cell junctions among the tumor cell soma (arrows), **(C)** dense core vesicles (arrows), **(D-E)** and processes and synapses (box). The synapses seen in *E2f5 TKO* tumors are amacrine-like synapses. **(F)** TEM of amacrine synapses (boxes) at the inner plexiform layer from a *Chx10-Cre;Rb$^{lox/lox}$; E2f5$^{lox/lox}$* (*E2f5 DKO)* retina shown as comparison of the amacrine-like synapses seen in retinoblastoma tumors.

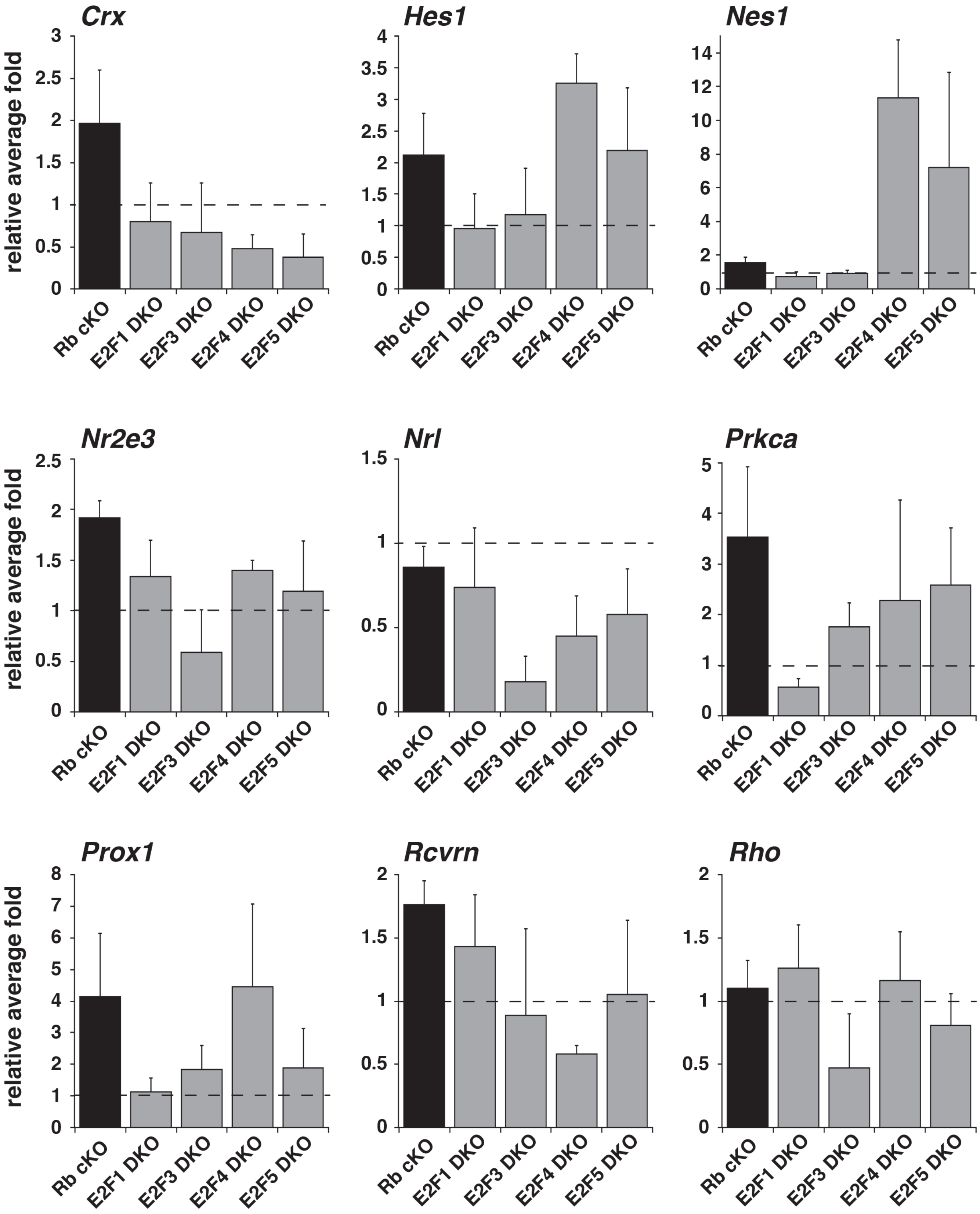


**Supplementary Figure S2: Real Time RT-PCR Analysis of Retinal Development Genes.** Real time RT-PCR analyses for P21 retinae from E2f DKO mice for various retinal development genes. Relative average fold was calculated compared to wt. Each bar is the mean and standard deviation of 5 biological replicates ran in duplicate.

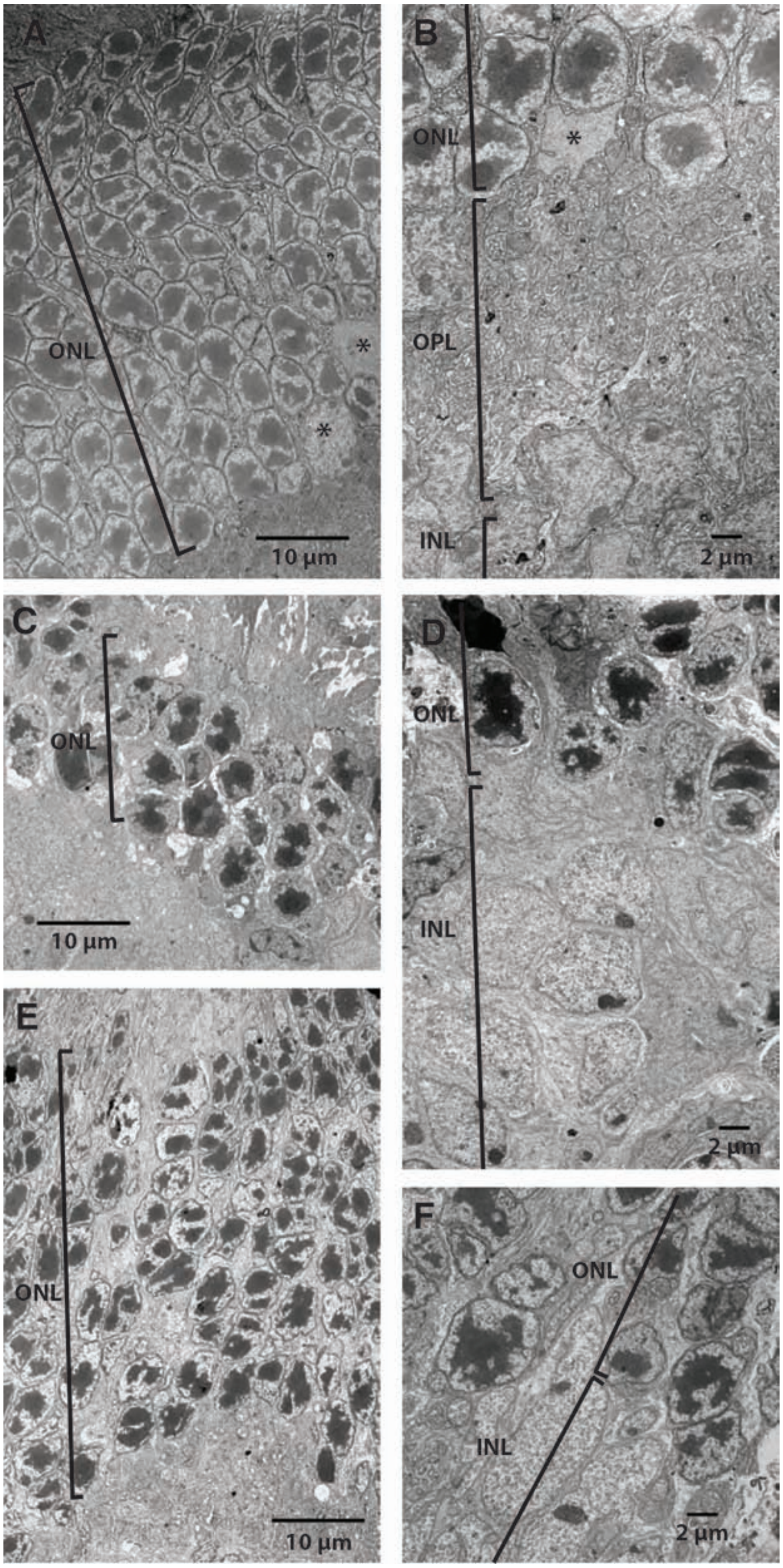


**Supplementary Figure S3: Transmission Electron Microscopy from E2f DKO Retinae.** **(A-F)** Transmission electron microscopy (TEM) from P21 E2f DKO retinae. **(A-B)** *Chx10-Cre;Rb*$^{lox/lox}$*;E2f1*$^{-/-}$ (*E2f1 DKO*) retinae show normal thickness of **(A)** the outer nuclear layer (ONL) and **(B)** outer plexiform layer (OPL) with some evidence of persistent progenitors (asterisk). **(C-D)** *Chx10-Cre;Rb*$^{lox/lox}$*;E2f3*$^{lox/lox}$ (*E2f3 DKO*) retinae show thinning of **(C)** the ONL and **(D)** OPL. **(E-F)** *Chx10-Cre;Rb*$^{lox/lox}$*;E2f5*$^{lox/lox}$ (*E2f5 DKO*) retinae also show thinning of the **(E)** ONL and **(F)** OPL. INL: inner nuclear layer.

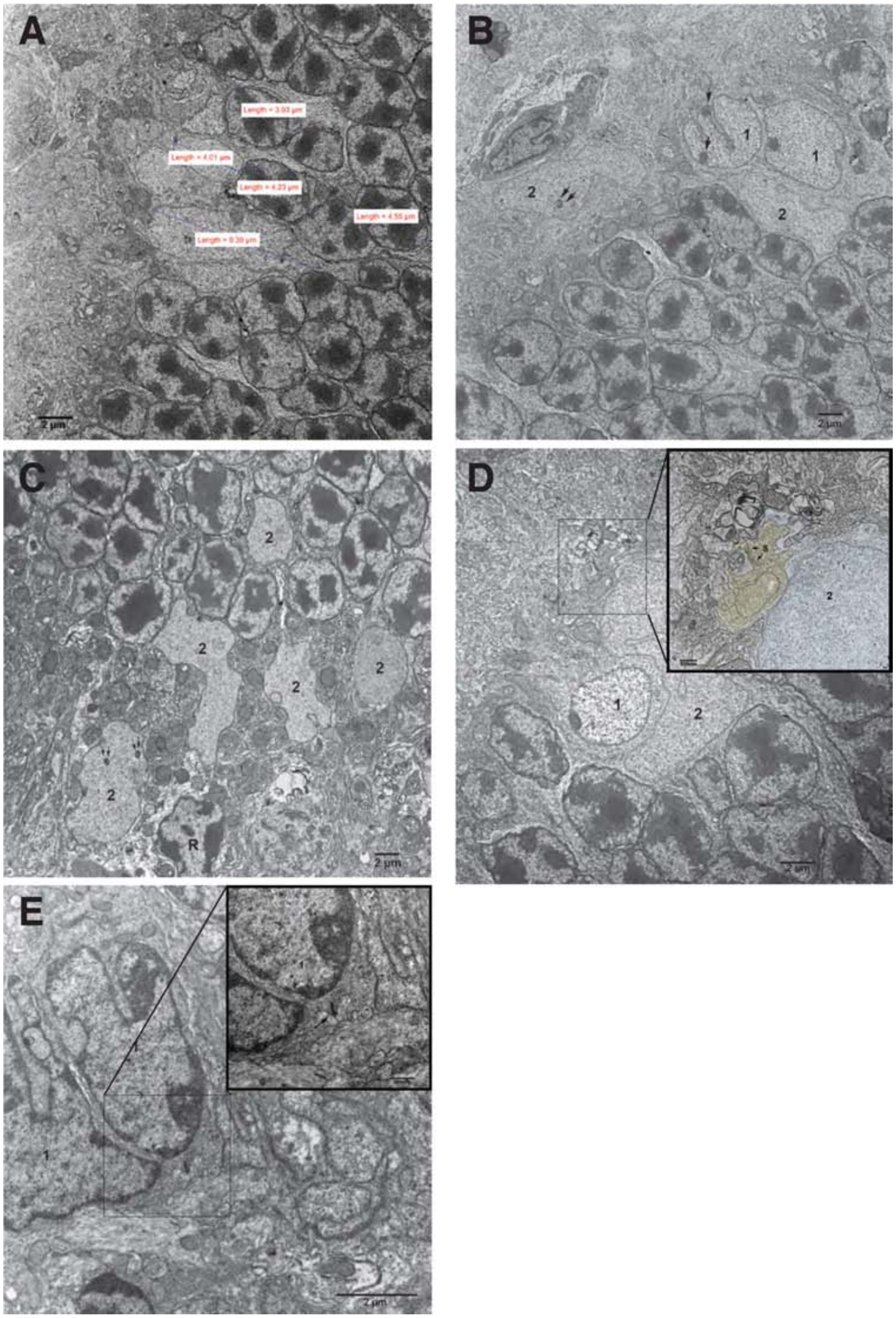


**Supplementary Figure S4: Transmission Electron Microscopy Showing Persistent Progenitor Cells in E2f TKO Retinae.** TEM analysis of E2f TKO retinae show **(A)** The "length" of unusual cells with pale nuclei termed "persistent progenitors" varies from the average diameter of normal rods to more than twice as large. Also, their chromatin is less condensed than normal rods. **(B)** The differences in the size and shape of these cells may reflect two stages. Stage 1 cells are approximately the size of normal rods but with some nuclear invagination. Their chromatin is more dispersed and some present nucleoli. Stage 2 cells are large in size, often elongated. Their nucleus is highly pleomorphic, with no evidence of chromatin condensation. No nucleoli are present but small dark circular inclusion bodies are often seen within the pale nucleus. Clumps of persistent progenitors in both stages are usually found on the proximal edge of the OPL within the ONL. **(C)** Both cell stages (stage2) and normal rods (R) can be displaced into the outer plexiform layer (OPL). Displaced rod somata are often elongated. **(D)** Example of stage 1 and stage 2 cell. Inset: Stage 2 cell could be traced, showing process projecting into OPL in close apposition to neighboring rod spherule. **(E)** Two stage 1 cells are shown, one appears to contain spherule elements: synaptic ribbon and junction with two horizontal cells.

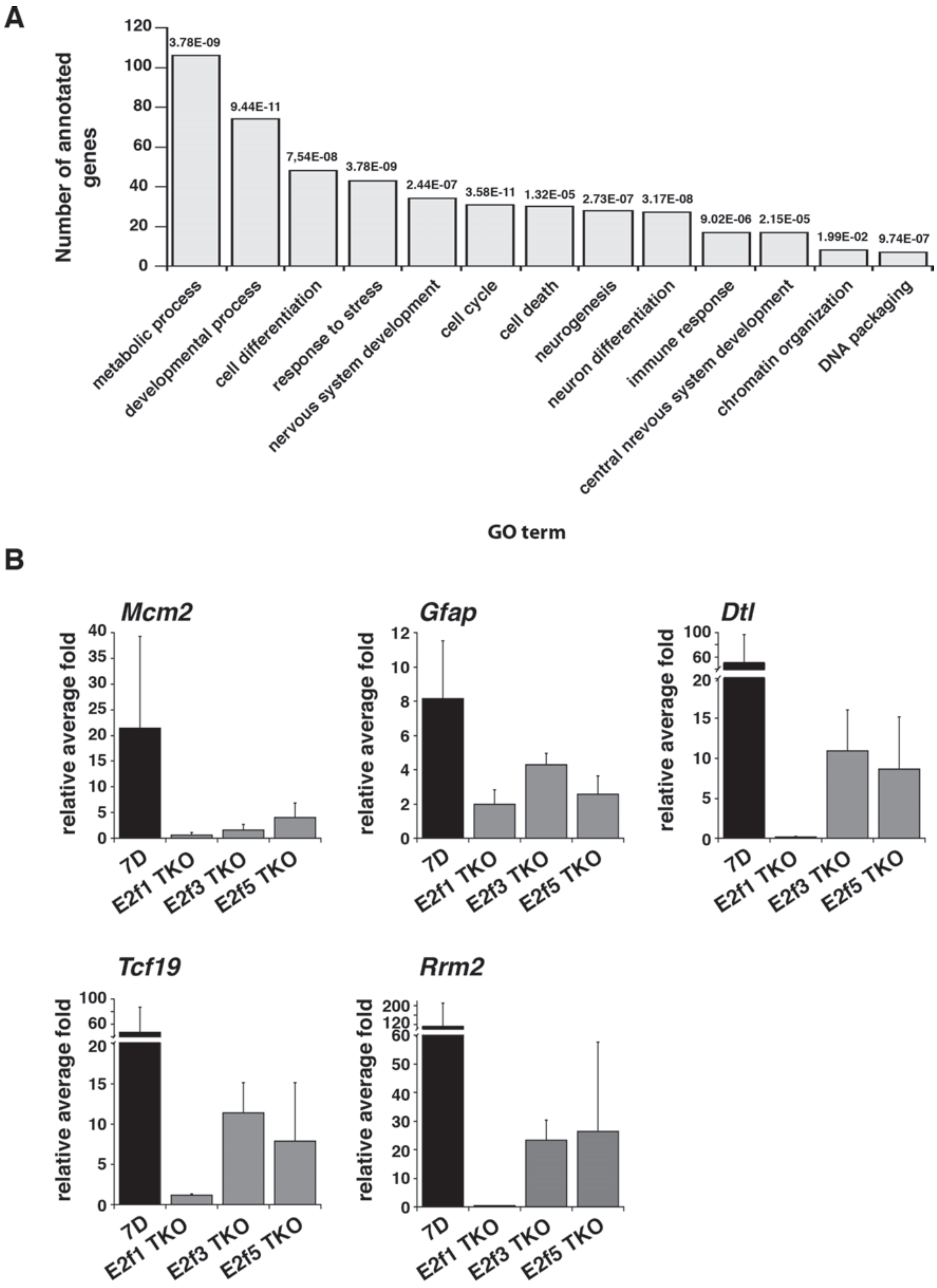


**Supplementary Figure S5: Real Time RT-PCR Validation of Putative Rb-Target Genes Involved in Retinal Development.** **(A)** Histogram from the Gene Ontology (GO) analysis of the 322 genes that are putative Rb-targets involved in retinal development. Each bar is the number of annotated genes from a subset of the GO terms found. Numbers above the bars depict the respective P-values. **(B)** Real time RT-PCR validation of a subset of genes involved in retinal development show a significant increase in gene expression in the *Chx10-Cre;Rb*$^{lox/lox}$*;p107*$^{-/-}$ (*7D*), *Chx10-Cre;Rb*$^{lox/lox}$*;p107*$^{-/-}$*;E2f3*$^{lox/lox}$ *(E2f3 TKO)* and *Chx10-Cre;Rb*$^{lox/lox}$*;p107*$^{-/-}$*;E2f5*$^{lox/lox}$ (*E2f5 TKO*) mouse strains that display retinal development defects but not in *Chx10-Cre;Rb*$^{lox/lox}$*;p107*$^{-/-}$*;E2f1*$^{-/-}$ *(E2f1 TKO)* which rescue the developmental defects. Each bar is the mean and standard deviation of 5 biological replicates ran in duplicate.

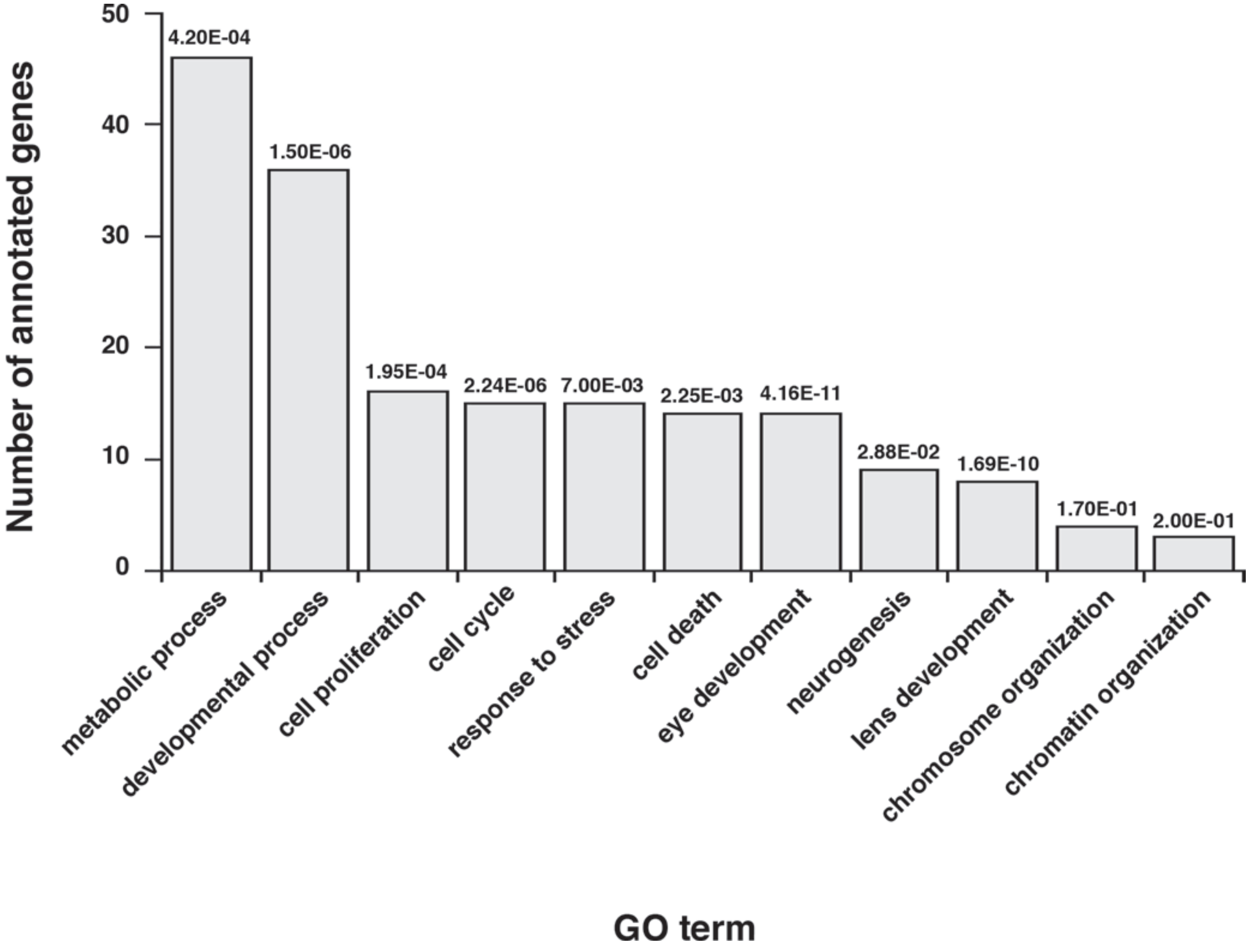


**Supplementary Figure S6: Gene Ontology Analysis of Putative Rb-Target Genes Involved in Retinoblastoma.** Histogram from the Gene Ontology (GO) analysis of the 148 genes that are putative Rb-targets involved in retinal tumorigenesis. Each bar is the number of annotated genes from a subset of the GO terms found. Numbers above the bars depict the respective P-values.

## Supplementary Table S1. Retinal Differentiation Gene List

| genesymbol | genetitle |
|---|---|
| 130004C03 | hypothetical LOC403343 |
| 1500011B03Rik | RIKEN cDNA 1500011B03 gene |
| 1500015O10Rik | RIKEN cDNA 1500015O10 gene |
| 1700007K13Rik | RIKEN cDNA 1700007K13 gene |
| 1700023H06Rik | RIKEN cDNA 1700023H06 gene |
| 2010011I20Rik | RIKEN cDNA 2010011I20 gene |
| 2010315B03Rik | RIKEN cDNA 2010315B03 gene |
| 2310067E19Rik | RIKEN cDNA 2310067E19 gene |
| 2610507I01Rik | RIKEN cDNA 2610507I01 gene |
| 2700089E24Rik | RIKEN cDNA 2700089E24 gene |
| 2810039B14Rik | RIKEN cDNA 2810039B14 gene |
| 2810055F11Rik | RIKEN cDNA 2810055F11 gene |
| 2900046D03Rik | RIKEN cDNA 2900046D03 gene |
| 2900052N01Rik | RIKEN cDNA 2900052N01 gene |
| 3110035E14Rik | RIKEN cDNA 3110035E14 gene |
| 4632432E15Rik | RIKEN cDNA 4632432E15 gene |
| 4833420G17Rik | RIKEN cDNA 4833420G17 gene |
| 4933406J10Rik | RIKEN cDNA 4933406J10 gene |
| 4933409K07Rik///Gm10590///Gm3893///Gm5859///Gm7819 | RIKEN cDNA 4933409K07 gene///predicted gene 10590///predicted gene 3893///predict |
| 5730408A14Rik | RIKEN cDNA 5730408A14 gene |
| 5730552O08Rik | RIKEN cDNA 5730552O08 gene |
| 5830443J22Rik | RIKEN cDNA 5830443J22 gene |
| 6030438J01 | hypothetical protein 6030438J01 |
| 6330418B08Rik | RIKEN cDNA 6330418B08 gene |
| 6530402F18Rik | RIKEN cDNA 6530402F18 gene |
| 6720422M22Rik | RIKEN cDNA 6720422M22 gene |
| 7120451J01Rik///LOC100503726 | RIKEN cDNA 7120451J01 gene///hypothetical protein LOC100503726 |
| 9230105E10Rik | RIKEN cDNA 9230105E10 gene |
| 9330161A08Rik | RIKEN cDNA 9330161A08 gene |
| 9430025M13Rik | RIKEN cDNA 9430025M13 gene |
| 9430065F17Rik | RIKEN cDNA 9430065F17 gene |
| 9630009C16 | hypothetical protein 9630009C16 |
| A030001D20Rik | RIKEN cDNA A030001D20 gene |
| A2m | alpha-2-macroglobulin |

(*Continued*)

| genesymbol | genetitle |
|---|---|
| A430105J06Rik | RIKEN cDNA A430105J06 gene |
| A930004D18Rik | RIKEN cDNA A930004D18 gene |
| A930028C08Rik | RIKEN cDNA A930028C08 gene |
| A930030B08Rik | RIKEN cDNA A930030B08 gene |
| A930036A04Rik | RIKEN cDNA A930036A04 gene |
| Aard | alanine and arginine rich domain containing protein |
| Abca9 | ATP-binding cassette, sub-family A (ABC1), member 9 |
| Adarb2 | adenosine deaminase, RNA-specific, B2 |
| Ahrr | aryl-hydrocarbon receptor repressor |
| AI835735 | expressed sequence AI835735 |
| Akap5 | A kinase (PRKA) anchor protein 5 |
| Ankrd33b | ankyrin repeat domain 33B |
| Antxr2 | anthrax toxin receptor 2 |
| Apobec1 | apolipoprotein B mRNA editing enzyme, catalytic polypeptide 1 |
| Apobec2 | apolipoprotein B mRNA editing enzyme, catalytic polypeptide 2 |
| Apoc1 | apolipoprotein C-I |
| Arhgap26 | Rho GTPase activating protein 26 |
| Arhgap31 | Rho GTPase activating protein 31 |
| Arsk | arylsulfatase K |
| Atad2 | ATPase family, AAA domain containing 2 |
| Atp1a2 | ATPase, Na+/K+ transporting, alpha 2 polypeptide |
| B2m | beta-2 microglobulin |
| BC023969 | cDNA sequence BC023969 |
| BC064078 | cDNA sequence BC064078 |
| Bcan | brevican |
| BE949265 | cDNA sequence BE949265 |
| Bmp2 | bone morphogenetic protein 2 |
| C030034I22Rik | RIKEN cDNA C030034I22 gene |
| C130071C03Rik | RIKEN cDNA C130071C03 gene |
| C1qa | complement component 1, q subcomponent, alpha polypeptide |
| C1qb | complement component 1, q subcomponent, beta polypeptide |
| C1qc | complement component 1, q subcomponent, C chain |
| C4b | complement component 4B (Childo blood group) |
| C4bp-ps1 | complement component 4 binding protein, pseudogene 1 |
| Carhsp1 | calcium regulated heat stable protein 1 |
| Ccdc24 | coiled-coil domain containing 24 |

(*Continued*)

| genesymbol | genetitle |
|---|---|
| Ccl21a///Ccl21b///Ccl21c///Gm10591///Gm13304///Gm1987///LOC100041593 | chemokine (C-C motif) ligand 21A (serine)///chemokine (C-C motif) ligand 21B (leucine) |
| Ccne2 | cyclin E2 |
| Cd44 | CD44 antigen |
| Cd53 | CD53 antigen |
| Cd59a | CD59a antigen |
| Cdc20 | cell division cycle 20 homolog (S. cerevisiae) |
| Cdca8 | cell division cycle associated 8 |
| Cdk1 | cyclin-dependent kinase 1 |
| Cdkn1a | cyclin-dependent kinase inhibitor 1A (P21) |
| Cdt1 | chromatin licensing and DNA replication factor 1 |
| Cenpf | centromere protein F |
| Cenpk | centromere protein K |
| Cep164 | centrosomal protein 164 |
| Chek1 | checkpoint kinase 1 homolog (S. pombe) |
| Chl1 | cell adhesion molecule with homology to L1CAM |
| Chrdl1 | chordin-like 1 |
| Ckap2 | cytoskeleton associated protein 2 |
| Cks1b | CDC28 protein kinase 1b |
| Cldn7 | claudin 7 |
| Clspn | claspin homolog (Xenopus laevis) |
| Col18a1 | collagen, type XVIII, alpha 1 |
| Col19a1 | collagen, type XIX, alpha 1 |
| Col1a2 | collagen, type I, alpha 2 |
| Col2a1 | collagen, type II, alpha 1 |
| Col9a3 | collagen, type IX, alpha 3 |
| Comt1 | catechol-O-methyltransferase 1 |
| Crhbp | corticotropin releasing hormone binding protein |
| Crym | crystallin, mu |
| Csrp2 | cysteine and glycine-rich protein 2 |
| Ctss | cathepsin S |
| Cxcl12 | chemokine (C-X-C motif) ligand 12 |
| Cxcl13 | chemokine (C-X-C motif) ligand 13 |
| Cxcl14 | chemokine (C-X-C motif) ligand 14 |
| D17H6S56E-5 | DNA segment, Chr 17, human D6S56E 5 |
| D4Wsu53e | DNA segment, Chr 4, Wayne State University 53, expressed |

(*Continued*)

| genesymbol | genetitle |
|---|---|
| Daam1 | dishevelled associated activator of morphogenesis 1 |
| Dctd | dCMP deaminase |
| Dcx | doublecortin |
| Dixdc1 | DIX domain containing 1 |
| Dleu7 | deleted in lymphocytic leukemia, 7 |
| Dnahc9 | dynein, axonemal, heavy chain 9 |
| Dtl | denticleless homolog (Drosophila) |
| Dusp12 | dual specificity phosphatase 12 |
| Edn2 | endothelin 2 |
| Elavl1 | ELAV (embryonic lethal, abnormal vision, Drosophila)-like 1 (Hu antigen R) |
| Emp1 | epithelial membrane protein 1 |
| Eno4 | enolase 4 |
| Eps8 | epidermal growth factor receptor pathway substrate 8 |
| Eps8l1 | EPS8-like 1 |
| Erc1 | ELKS/RAB6-interacting/CAST family member 1 |
| Fam107b | family with sequence similarity 107, member B |
| Fam111a | family with sequence similarity 111, member A |
| Fam129a | family with sequence similarity 129, member A |
| Fam129a | family with sequence similarity 129, member A |
| Fam5c | family with sequence similarity 5, member C |
| Fbln1 | fibulin 1 |
| Fbn2 | fibrillin 2 |
| Fcgr3 | Fc receptor, IgG, low affinity III |
| Fignl1 | fidgetin-like 1 |
| Fscn2 | fascin homolog 2, actin-bundling protein, retinal (Strongylocentrotus purpuratus) |
| Fyco1 | FYVE and coiled-coil domain containing 1 |
| Gabrb3 | gamma-aminobutyric acid (GABA) A receptor, subunit beta 3 |
| Gas5 | growth arrest specific 5 |
| Gas7 | growth arrest specific 7 |
| Gbp10///Mpa2l | guanylate-binding protein 10///macrophage activation 2 like |
| Gbp6 | guanylate binding protein 6 |
| Gckr | glucokinase regulatory protein |
| Gdpd3 | glycerophosphodiester phosphodiesterase domain containing 3 |
| Gfap | glial fibrillary acidic protein |
| Gins1 | GINS complex subunit 1 (Psf1 homolog) |
| Gja1 | gap junction protein, alpha 1 |
| Glb1l3 | galactosidase, beta 1 like 3 |

(*Continued*)

| genesymbol | genetitle |
|---|---|
| Gm10037 | predicted gene 10037 |
| Gm10393///Plac9 | predicted gene 10393///placenta specific 9 |
| Gm11276///Hist1h2ao | predicted gene 11276///histone cluster 1, H2ao |
| Gm12758 | predicted gene 12758 |
| Gm2115 | predicted gene 2115 |
| Gm3893///Gm5859///Gm7819 | predicted gene 3893///predicted pseudogene 5859///predicted gene 7819 |
| Gm7120 | predicted gene 7120 |
| Gmcl1 | germ cell-less homolog 1 (Drosophila) |
| Gpc3 | glypican 3 |
| Gpr137b | G protein-coupled receptor 137B |
| Gpr34 | G protein-coupled receptor 34 |
| Grik1 | glutamate receptor, ionotropic, kainate 1 |
| Grm3 | glutamate receptor, metabotropic 3 |
| Guca1b | guanylate cyclase activator 1B |
| H2-T10///H2-T22///H2-T9 | histocompatibility 2, T region locus 10///histocompatibility 2, T region locus 22 |
| H2afj | H2A histone family, member J |
| Hcls1 | hematopoietic cell specific Lyn substrate 1 |
| Hddc3 | HD domain containing 3 |
| Heatr1 | HEAT repeat containing 1 |
| Hebp1 | heme binding protein 1 |
| Hells | helicase, lymphoid specific |
| Heph | hephaestin |
| Herc6 | hect domain and RLD 6 |
| Hist1h2be | Histone cluster 1, H2be |
| Ifit2 | interferon-induced protein with tetratricopeptide repeats 2 |
| Ifitm3 | interferon induced transmembrane protein 3 |
| Igfbpl1 | insulin-like growth factor binding protein-like 1 |
| Ildr2 | immunoglobulin-like domain containing receptor 2 |
| Insl5 | insulin-like 5 |
| Irf3 | interferon regulatory factor 3 |
| Kbtbd11 | kelch repeat and BTB (POZ) domain containing 11 |
| Kcne1l | potassium voltage-gated channel, Isk-related family, member 1-like, pseudogene |
| Klf2 | Kruppel-like factor 2 (lung) |
| Krt24 | keratin 24 |
| Laptm5 | lysosomal-associated protein transmembrane 5 |
| Lars2 | leucyl-tRNA synthetase, mitochondrial |

(*Continued*)

| genesymbol | genetitle |
|---|---|
| Lgi3 | leucine-rich repeat LGI family, member 3 |
| LOC630164///Xlr3a///Xlr3b///Xlr3c | x-linked lymphocyte-regulated protein 3B-like///X-linked lymphocyte-regulated 3A /// |
| LOC676974 | glucose-6-phosphate isomerase-like |
| Ltbp1 | latent transforming growth factor beta binding protein 1 |
| Lyplal1 | lysophospholipase-like 1 |
| Lyz1 | lysozyme 1 |
| Lztfl1 | leucine zipper transcription factor-like 1 |
| Madd | MAP-kinase activating death domain |
| Man2a2 | mannosidase 2, alpha 2 |
| Mapk6 | mitogen-activated protein kinase 6 |
| March6 | membrane-associated ring finger (C3HC4) 6 |
| Matn2 | matrilin 2 |
| Mcm2 | minichromosome maintenance deficient 2 mitotin (S. cerevisiae) |
| Mcm3 | minichromosome maintenance deficient 3 (S. cerevisiae) |
| Mcm5 | minichromosome maintenance deficient 5, cell division cycle 46 (S. cerevisiae) |
| Mcm6 | minichromosome maintenance deficient 6 (MIS5 homolog, S. pombe) (S. cerevisiae) |
| Mcm7 | minichromosome maintenance deficient 7 (S. cerevisiae) |
| Mcoln1 | mucolipin 1 |
| Mdk | midkine |
| Mef2c | myocyte enhancer factor 2C |
| Mki67 | antigen identified by monoclonal antibody Ki 67 |
| Mpeg1 | macrophage expressed gene 1 |
| Mt2 | metallothionein 2 |
| Myb | myeloblastosis oncogene |
| Mybl1 | myeloblastosis oncogene-like 1 |
| Mycl1 | v-myc myelocytomatosis viral oncogene homolog 1, lung carcinoma derived (avian) |
| Myo7a | myosin VIIA |
| Ncaph | non-SMC condensin I complex, subunit H |
| Ndel1 | nuclear distribution gene E-like homolog 1 (A. nidulans) |
| Ndufa12 | NADH dehydrogenase (ubiquinone) 1 alpha subcomplex, 12 |
| Neurog2 | neurogenin 2 |
| Nfib | nuclear factor I/B |
| Nfix | nuclear factor I/X |
| Nmral1 | NmrA-like family domain containing 1 |
| Nnat | neuronatin |
| Nrip2 | nuclear receptor interacting protein 2 |
| Nusap1 | nucleolar and spindle associated protein 1 |

(*Continued*)

| genesymbol | genetitle |
|---|---|
| Ogn | osteoglycin |
| Opn1sw | opsin 1 (cone pigments), short-wave-sensitive (color blindness, tritan) |
| P2ry12 | purinergic receptor P2Y, G-protein coupled 12 |
| Paip1 | polyadenylate binding protein-interacting protein 1 |
| Parp3 | poly (ADP-ribose) polymerase family, member 3 |
| Pbk | PDZ binding kinase |
| Pcolce | procollagen C-endopeptidase enhancer protein |
| Pcp2 | Purkinje cell protein 2 (L7) |
| Pde3b | phosphodiesterase 3B, cGMP-inhibited |
| Pde6h | phosphodiesterase 6H, cGMP-specific, cone, gamma |
| Pdzrn3 | PDZ domain containing RING finger 3 |
| Pex19 | peroxisomal biogenesis factor 19 |
| Pisd-ps3 | phosphatidylserine decarboxylase, pseudogene 3 |
| Pitpnm3 | PITPNM family member 3 |
| Pla2g16 | phospholipase A2, group XVI |
| Plcd3 | phospholipase C, delta 3 |
| Plekhf1 | pleckstrin homology domain containing, family F (with FYVE domain) member 1 |
| Pnpla3 | patatin-like phospholipase domain containing 3 |
| Pole2 | polymerase (DNA directed), epsilon 2 (p59 subunit) |
| Ppara | peroxisome proliferator activated receptor alpha |
| Ppcdc | phosphopantothenoylcysteine decarboxylase |
| Ppic | peptidylprolyl isomerase C |
| Ppox | protoporphyrinogen oxidase |
| Ppp1r12b | protein phosphatase 1, regulatory (inhibitor) subunit 12B |
| Prc1 | protein regulator of cytokinesis 1 |
| Prcp | prolylcarboxypeptidase (angiotensinase C) |
| Prelp | proline arginine-rich end leucine-rich repeat |
| Prim1 | DNA primase, p49 subunit |
| Prkca | protein kinase C, alpha |
| Prph2 | peripherin 2 |
| Psma3 | Proteasome (prosome, macropain) subunit, alpha type 3 |
| Ptprz1 | protein tyrosine phosphatase, receptor type Z, polypeptide 1 |
| Ptx3 | pentraxin related gene |
| Pycard | PYD and CARD domain containing |
| Qars | glutaminyl-tRNA synthetase |
| Rab31 | RAB31, member RAS oncogene family |
| Rabgef1 | RAB guanine nucleotide exchange factor (GEF) 1 |

(*Continued*)

| genesymbol | genetitle |
|---|---|
| Rcn2 | reticulocalbin 2 |
| Retn | resistin |
| Rgs13 | regulator of G-protein signaling 13 |
| Rnase4 | ribonuclease, RNase A family 4 |
| Rnf207 | ring finger protein 207 |
| Rpgrip1 | retinitis pigmentosa GTPase regulator interacting protein 1 |
| Rprd1b | regulation of nuclear pre-mRNA domain containing 1B |
| Rps25 | ribosomal protein S25 |
| Rps9 | ribosomal protein S9 |
| Rrm2 | ribonucleotide reductase M2 |
| S100a4 | S100 calcium binding protein A4 |
| S100a6 | S100 calcium binding protein A6 (calcyclin) |
| Scn2b | sodium channel, voltage-gated, type II, beta |
| Scn7a | sodium channel, voltage-gated, type VII, alpha |
| Sdha | succinate dehydrogenase complex, subunit A, flavoprotein (Fp) |
| Sec22a | SEC22 vesicle trafficking protein homologe A (S. cerevisiae) |
| Serinc3 | serine incorporator 3 |
| Serping1 | serine (or cysteine) peptidase inhibitor, clade G, member 1 |
| Sh2d1a | SH2 domain protein 1A |
| Slc13a4 | solute carrier family 13 (sodium/sulfate symporters), member 4 |
| Slc38a6 | solute carrier family 38, member 6 |
| Slco4a1 | solute carrier organic anion transporter family, member 4a1 |
| Smc2 | structural maintenance of chromosomes 2 |
| Socs2 | suppressor of cytokine signaling 2 |
| Sox11 | SRY-box containing gene 11 |
| Sox4 | SRY-box containing gene 4 |
| Spc25 | SPC25, NDC80 kinetochore complex component, homolog (S. cerevisiae) |
| Srp54b | signal recognition particle 54B |
| Srpk2 | serine/arginine-rich protein specific kinase 2 |
| Stard7 | START domain containing 7 |
| Stk17b | serine/threonine kinase 17b (apoptosis-inducing) |
| Suclg2 | succinate-Coenzyme A ligase, GDP-forming, beta subunit |
| Syne2 | synaptic nuclear envelope 2 |
| Sys1 | SYS1 Golgi-localized integral membrane protein homolog (S. cerevisiae) |
| Tcf19 | transcription factor 19 |
| Tcfap2c | transcription factor AP-2, gamma |
| Tctn3 | tectonic family member 3 |

(*Continued*)

| genesymbol | genetitle |
|---|---|
| Tgm2 | transglutaminase 2, C polypeptide |
| Tia1 | cytotoxic granule-associated RNA binding protein 1 |
| Timm44 | translocase of inner mitochondrial membrane 44 |
| Tmem150c | transmembrane protein 150C |
| Tmlhe | trimethyllysine hydroxylase, epsilon |
| Tmx4 | thioredoxin-related transmembrane protein 4 |
| Tnfrsf21 | tumor necrosis factor receptor superfamily, member 21 |
| Tnnt1 | troponin T1, skeletal, slow |
| Top2a | topoisomerase (DNA) II alpha |
| Trim59 | tripartite motif-containing 59 |
| Tspan6 | tetraspanin 6 |
| Ttc21a | Tetratricopeptide repeat domain 21A |
| Tube1 | epsilon-tubulin 1 |
| Tyrobp | TYRO protein tyrosine kinase binding protein |
| Ube2c | ubiquitin-conjugating enzyme E2C |
| Ube2m | ubiquitin-conjugating enzyme E2M (UBC12 homolog, yeast) |
| Uhrf1 | ubiquitin-like, containing PHD and RING finger domains, 1 |
| Ung | uracil DNA glycosylase |
| Vamp3 | vesicle-associated membrane protein 3 |
| Vax2os1 | Vax2 opposite strand transcript 1 |
| Vcan | versican |
| Vmn2r29 | vomeronasal 2, receptor 29 |
| Vps13b | vacuolar protein sorting 13B (yeast) |
| Wdfy1 | WD repeat and FYVE domain containing 1 |
| Wdr89 | WD repeat domain 89 |
| Wisp1 | WNT1 inducible signaling pathway protein 1 |
| Wnt5a | wingless-related MMTV integration site 5A |
| Xaf1 | XIAP associated factor 1 |
| Zcchc24 | zinc finger, CCHC domain containing 24 |
| Zfp420 | zinc finger protein 420 |
| Zfp874a | zinc finger protein 874a |
| Zic1 | zinc finger protein of the cerebellum 1 |
| Zic5 | zinc finger protein of the cerebellum 5 |

## Supplementary Table S2. Tumorigenesis Gene List

| genesymbol | genetitle | Epigenetic | Deregulated in human RB |
|---|---|---|---|
| 1190002F15Rik | RIKEN cDNA 1190002F15 gene | No | No |
| 1200003I10Rik///1200015M12Rik///A130040M12Rik///E430024C06Rik | RIKEN cDNA 1200003I10 gene///RIKEN cDNA 1200015M12 gene///RIKEN cDNA A130040M12 gen | No | No |
| 1200015M12Rik///A130040M12Rik///E430024C06Rik | RIKEN cDNA 1200015M12 gene///RIKEN cDNA A130040M12 gene///RIKEN cDNA E430024C06 gen | No | No |
| 2810417H13Rik | RIKEN cDNA 2810417H13 gene | Yes | No |
| 3110003A17Rik | RIKEN cDNA 3110003A17 gene | No | No |
| 4833408G04Rik | RIKEN cDNA 4833408G04 gene | No | No |
| 5330426P16Rik | RIKEN cDNA 5330426P16 gene | No | No |
| 9030411K21Rik | RIKEN cDNA 9030411K21 gene | No | No |
| 9430047L24Rik | RIKEN cDNA 9430047L24 gene | No | No |
| 9530085L11Rik | RIKEN cDNA 9530085L11 gene | No | No |
| A330094K24Rik | RIKEN cDNA A330094K24 gene | No | No |
| A530083I20Rik | RIKEN cDNA A530083I20 gene | No | No |
| Abcb1a | ATP-binding cassette, sub-family B (MDR/TAP), member 1A | No | No |
| Abcc9 | ATP-binding cassette, sub-family C (CFTR/MRP), member 9 | No | No |
| Abce1 | ATP-binding cassette, sub-family E (OABP), member 1 | No | No |
| Acaa1a///Acaa1b | acetyl-Coenzyme A acyltransferase 1A///acetyl-Coenzyme A acyltransferase 1B | No | No |
| Agpat3 | 1-acylglycerol-3-phosphate O-acyltransferase 3 | Yes | Yes |
| Aipl1 | aryl hydrocarbon receptor-interacting protein-like 1 | Yes | No |
| Akna | AT-hook transcription factor | No | Yes |
| Ankrd33 | ankyrin repeat domain 33 | No | Yes |
| Anln | anillin, actin binding protein | Yes | No |
| Arhgap11a | Rho GTPase activating protein 11A | Yes | No |
| Armc9 | armadillo repeat containing 9 | Yes | Yes |
| Aspm | asp (abnormal spindle)-like, microcephaly associated (Drosophila) | Yes | No |
| BC019943 | cDNA sequence BC019943 | No | No |
| BC059841 | cDNA sequence BC059841 | No | No |
| Bfsp1 | beaded filament structural protein 1, in lens-CP94 | No | No |
| Bub1 | budding uninhibited by benzimidazoles 1 homolog (S. cerevisiae) | Yes | No |
| Car8 | carbonic anhydrase 8 | No | No |
| Ccnb1 | cyclin B1 | Yes | No |
| Cd24a | CD24a antigen | No | No |
| Cdc20 | cell division cycle 20 homolog (S. cerevisiae) | Yes | No |

(*Continued*)

| genesymbol | genetitle | Epigenetic | Deregulated in human RB |
|---|---|---|---|
| Cdc6 | cell division cycle 6 homolog (S. cerevisiae) | Yes | No |
| Cdca3 | cell division cycle associated 3 | Yes | No |
| Cdca7 | cell division cycle associated 7 | No | No |
| Cdca7l | cell division cycle associated 7 like | Yes | No |
| Cdk2 | cyclin-dependent kinase 2 | Yes | No |
| Cds1 | CDP-diacylglycerol synthase 1 | No | No |
| Cks2 | CDC28 protein kinase regulatory subunit 2 | Yes | No |
| Cnnm4 | cyclin M4 | No | Yes |
| Col24a1 | collagen, type XXIV, alpha 1 | No | No |
| Col6a3 | collagen, type VI, alpha 3 | No | No |
| Crocc | ciliary rootlet coiled-coil, rootletin | Yes | No |
| Cryaa | crystallin, alpha A | No | No |
| Cryab | crystallin, alpha B | Yes | No |
| Cryba1 | crystallin, beta A1 | Yes | No |
| Cryba2 | crystallin, beta A2 | No | No |
| Cryba4 | crystallin, beta A4 | Yes | No |
| Crybb1 | crystallin, beta B1 | No | No |
| Crybb2 | crystallin, beta B2 | No | No |
| Crybb3 | crystallin, beta B3 | No | No |
| Cryga | crystallin, gamma A | No | No |
| Crygb | crystallin, gamma B | No | No |
| Crygc | crystallin, gamma C | No | No |
| Crygd | crystallin, gamma D | No | Yes |
| Crygf | crystallin, gamma F | No | No |
| Crygn | crystallin, gamma N | No | No |
| Crygs | crystallin, gamma S | No | No |
| D17H6S56E-5 | DNA segment, Chr 17, human D6S56E 5 | Yes | No |
| Dcx | doublecortin | Yes | Yes |
| Ddx6 | DEAD (Asp-Glu-Ala-Asp) box polypeptide 6 | No | Yes |
| Dnajc28 | DnaJ (Hsp40) homolog, subfamily C, member 28 | No | No |
| Dohh/// LOC100503793 | deoxyhypusine hydroxylase/monooxygenase///hypothetical protein LOC100503793 | No | No |
| Dpf3 | D4, zinc and double PHD fingers, family 3 | No | No |
| Dyx1c1 | dyslexia susceptibility 1 candidate 1 homolog (human) | No | No |
| E2f7 | E2F transcription factor 7 | No | No |
| E2f8 | E2F transcription factor 8 | Yes | No |
| Eno4 | enolase 4 | No | No |

(*Continued*)

| genesymbol | genetitle | Epigenetic | Deregulated in human RB |
|---|---|---|---|
| Fabp5 | fatty acid binding protein 5, epidermal | No | No |
| Fabp5///Gm3601 | fatty acid binding protein 5, epidermal///predicted gene 3601 | No | No |
| Fabp7 | fatty acid binding protein 7, brain | No | Yes |
| Fam161a | family with sequence similarity 161, member A | No | No |
| Fbxo5 | F-box protein 5 | Yes | No |
| Flad1///Lenep | RFad1, flavin adenine dinucleotide synthetase, homolog (yeast)/// lens epithelial prot | No | No |
| Gas2l3 | growth arrest-specific 2 like 3 | Yes | Yes |
| Giyd2 | GIY-YIG domain containing 2 | Yes | No |
| Gm7659 | Predicted gene 7659 | No | No |
| Gm9918 | predicted gene 9918 | No | No |
| Gpr116 | G protein-coupled receptor 116 | No | Yes |
| H2-Q6///LOC68395 | histocompatibility 2, Q region locus 6///histocompatibility 2, Q region locus 6-like | No | No |
| H2-Q7 | histocompatibility 2, Q region locus 7 | No | No |
| H2-T24 | histocompatibility 2, T region locus 24 | No | No |
| Hells | helicase, lymphoid specific | Yes | No |
| Hk2 | hexokinase 2 | No | No |
| Impg1 | interphotoreceptor matrix proteoglycan 1 | No | No |
| Jam2 | junction adhesion molecule 2 | No | Yes |
| Katnal2 | katanin p60 subunit A-like 2 | No | No |
| Kcnj14 | potassium inwardly-rectifying channel, subfamily J, member 14 | No | No |
| Lim2 | lens intrinsic membrane protein 2 | No | No |
| LOC100503456 | hypothetical LOC100503456 | No | No |
| LOC100505359///Xlr4a///Xlr4b///Xlr4c | x-linked lymphocyte-regulated protein 3A-like///X-linked lymphocyte-regulated 4A /// | No | No |
| Lrit2 | leucine-rich repeat, immunoglobulin-like and transmembrane domains 2 | No | No |
| Lrp2bp | Lrp2 binding protein | No | No |
| Lrrc67 | leucine rich repeat containing 67 | No | No |
| Mcm4 | minichromosome maintenance deficient 4 homolog (S. cerevisiae) | No | No |
| Mcm7 | minichromosome maintenance deficient 7 (S. cerevisiae) | Yes | No |
| Meig1 | meiosis expressed gene 1 | No | No |
| Ncapg2 | non-SMC condensin II complex, subunit G2 | No | Yes |
| Nfya | nuclear transcription factor-Y alpha | No | No |
| Nsg2 | neuron specific gene family member 2 | No | No |
| Nupr1 | nuclear protein 1 | No | No |

(*Continued*)

| genesymbol | genetitle | Epigenetic | Deregulated in human RB |
|---|---|---|---|
| Nxnl1 | nucleoredoxin-like 1 | No | No |
| Pde6a | phosphodiesterase 6A, cGMP-specific, rod, alpha | No | No |
| Pdia5 | protein disulfide isomerase associated 5 | No | Yes |
| Pfkl | phosphofructokinase, liver, B-type | No | Yes |
| Pigo | phosphatidylinositol glycan anchor biosynthesis, class O | No | No |
| Pim1 | proviral integration site 1 | Yes | No |
| Pla2r1 | phospholipase A2 receptor 1 | No | No |
| Pole | polymerase (DNA directed), epsilon | Yes | No |
| Pon1 | paraoxonase 1 | No | No |
| Prdx4 | peroxiredoxin 4 | Yes | No |
| Racgap1 | Rac GTPase-activating protein 1 | Yes | No |
| Rad51ap1 | RAD51 associated protein 1 | Yes | No |
| Rbm39 | RNA binding motif protein 39 | No | No |
| Rgr | retinal G protein coupled receptor | No | No |
| Rgs20 | regulator of G-protein signaling 20 | No | No |
| Rora | RAR-related orphan receptor alpha | No | No |
| Rs1 | retinoschisis (X-linked, juvenile) 1 (human) | No | Yes |
| Rspo2 | R-spondin 2 homolog (Xenopus laevis) | Yes | Yes |
| Samd7 | sterile alpha motif domain containing 7 | No | No |
| Sec22c | SEC22 vesicle trafficking protein homolog C (S. cerevisiae) | No | No |
| Serpina3n | serine (or cysteine) peptidase inhibitor, clade A, member 3N | No | No |
| Sgcg | sarcoglycan, gamma (dystrophin-associated glycoprotein) | No | No |
| Shcbp1 | Shc SH2-domain binding protein 1 | Yes | No |
| Slc17a1 | solute carrier family 17 (sodium phosphate), member 1 | No | No |
| Slc24a1 | solute carrier family 24 (sodium/potassium/calcium exchanger), member 1 | No | No |
| Slco4a1 | solute carrier organic anion transporter family, member 4a1 | No | Yes |
| Socs3 | suppressor of cytokine signaling 3 | No | No |
| Sox4 | SRY-box containing gene 4 | No | Yes |
| Spata1 | spermatogenesis associated 1 | Yes | No |
| Stk36 | serine/threonine kinase 36 (fused homolog, Drosophila) | No | No |
| Stxbp1 | syntaxin binding protein 1 | No | No |
| Synpo2 | synaptopodin 2 | No | No |
| Tek | endothelial-specific receptor tyrosine kinase | No | No |
| Tm4sf1 | transmembrane 4 superfamily member 1 | Yes | Yes |
| Tmlhe | trimethyllysine hydroxylase, epsilon | No | Yes |
| Topbp1 | topoisomerase (DNA) II binding protein 1 | Yes | No |

(*Continued*)

| genesymbol | genetitle | Epigenetic | Deregulated in human RB |
|---|---|---|---|
| Traf3ip3 | TRAF3 interacting protein 3 | No | No |
| Ube2t | ubiquitin-conjugating enzyme E2T (putative) | Yes | No |
| Ubtd2 | ubiquitin domain containing 2 | No | Yes |
| Ubxn7 | UBX domain protein 7 | No | No |
| Vtn | vitronectin | Yes | Yes |
| Wdr31 | WD repeat domain 31 | Yes | No |
| Wdr47 | WD repeat domain 47 | No | No |
| Xiap | X-linked inhibitor of apoptosis | No | No |
| Ypel2 | yippee-like 2 (Drosophila) | No | Yes |
| Zfp882 | zinc finger protein 882 | No | No |
| Zik1 | zinc finger protein interacting with K protein 1 | No | No |
